# Concordance and prognostic value of bone–marrow MRD and PET–CT in multiple myeloma: a systematic review and meta–analysis

**DOI:** 10.1016/j.eclinm.2026.103868

**Published:** 2026-04-06

**Authors:** Max Mendez–Lopez, Marco Talarico, Christoph Driessen

**Affiliations:** a Department of Medical Oncology/Haematology and Laboratory for Experimental Hematology, HOCH Ostschweiz, St Gallen, Switzerland; b IRCCS Azienda Ospedaliero – Universitaria di Bologna, Istituto di Ematologia “Seràgnoli”, Bologna, Italy; c Dipartimento di Scienze Mediche e Chirurgiche, Università di Bologna, Italy

**Keywords:** Multiple myeloma, Minimal residual disease, PET–CT, Concordance, Prognosis, Meta-analysis

## Abstract

**Background:**

Bone–marrow minimal residual disease (MRD) and positron emission tomography/computed tomography (PET–CT) are central to response assessment in multiple myeloma (MM); however, their agreement, discordant patterns, and how their joint results relate to progression–free survival have not been systematically quantified.

**Methods:**

We conducted a systematic review and random–effects meta–analysis, and searched PubMed and Cochrane CENTRAL from Jan 2015 to Sept 30, 2025, with an updated search to Jan 31, 2026. We included studies reporting paired MRD and PET–CT assessments, and, when available, progression–free survival (PFS), in patients with MM. Study–level 2 × 2 MRD/PET–CT tables (each test positive or negative) were abstracted or reconstructed from published counts or percentages. Primary outcomes were (1) cross–modality agreement (observed agreement and Cohen's κ) and directional discordance (log–odds of MRD^–^/PET–CT^+^ vs MRD^+^/PET–CT^−^), and (2) the prognostic effect of dual negativity (MRD^–^/PET–CT^−^ vs all other combinations) on PFS. We used random–effects models (REML with Hartung–Knapp adjustment) to estimate pooled hazard ratios for PFS. Sensitivity analyses were informed by QUADAS–2 (Quality Assessment of Diagnostic Accuracy Studies 2) and QUIPS (Quality in Prognosis Studies) risk–of–bias assessments. This study was registered with Open Science Framework (OSF; DOI 10.17605/OSF.IO/3CH9E).

**Findings:**

Ten cohorts contributed 1138 paired MRD/PET–CT assessments. The joint 2 × 2 distribution was: (a) MRD^–^/PET–CT^+^ 145 (12.7%), (b) MRD^−^/PET–CT^−^ 499 (43.8%), (c) MRD^+^/PET–CT^−^ 310 (27.2%), and (d) MRD^+^/PET–CT^+^ 184 (16.2%). Observed agreement was 60.0%, and pooled κ was 0.14 (95% CI 0.03–0.25; I^2^ 57%). Discordant results predominantly reflected MRD^+^/PET–CT^−^ rather than MRD^–^/PET–CT^+^ (primary pooled log–odds −0.83, 95% CI −1.87 to 0.21; I^2^ 93%; k = 10). In five studies reporting PFS by joint MRD/PET–CT status, dual negativity was associated with substantially longer PFS than all other categories (pooled HR 0.34 (95% CI 0.22–0.51); τ^2^ 0.03; I^2^ 27%; k = 5).

**Interpretation:**

MRD and PET–CT interrogate distinct but complementary disease compartments, yielding low statistical concordance yet strong joint prognostic value. Dual MRD/PET–CT negativity reproducibly identifies a low–risk subgroup, whereas discordant patterns capture biologically heterogeneous residual disease. Prospective myeloma trials should prespecify all four MRD/PET–CT categories, align assessment timing and thresholds, and routinely report concordance and discordance to enable response–adapted treatment.

**Funding:**

None.

**Research in context**

**Evidence before this study**

Bone–marrow minimal residual disease (MRD) negativity and PET–CT negativity are each established prognostic markers in multiple myeloma (MM), but their cross–modality concordance and combined prognostic effect have not been quantitatively synthesised. Reports from contemporary trials and observational cohorts suggest incomplete overlap between MRD and imaging responses, consistent with complementary biological information, but no prior meta-analysis has jointly quantified concordance/discordance and the prognostic impact of dual negativity. We searched PubMed and Cochrane CENTRAL for studies published between Jan 1, 2015, and April 30, 2025 (with protocol-amended targeted searches up to Sept 30, 2025 to capture late 2025 reports) using MeSH terms and keywords for multiple myeloma, minimal residual disease, and PET or PET–CT, without language restrictions (full strategies in Supplementary Methods). Following peer review, we updated the search through Jan 31, 2026 using the same strategy. We also screened reference lists, prior systematic reviews, and conference abstracts.

**Added value of this study**

This is, to our knowledge, the first systematic review and meta–analysis to quantify both cross–modality agreement (including the direction of discordance) and the prognostic effect of joint MRD/PET–CT results in multiple myeloma. We show that chance–corrected concordance between MRD and PET–CT is consistently low despite high individual prognostic value, and that dual negativity defines a robust low–risk subgroup across diverse treatment schemes and MRD/PET–CT methodologies.

**Implications of all the available evidence**

Taken together, current evidence supports MRD and PET–CT as complementary rather than interchangeable tools. Dual negativity captures a biologically favourable phenotype with substantially reduced progression risk, whereas discordant states signal persistent disease in either marrow or extramedullary compartments. Future trials and registries should mandate joint MRD/PET–CT reporting, standardise time–points and thresholds, and evaluate response–adapted strategies that explicitly incorporate concordant and discordant categories.

## Introduction

Minimal residual disease (MRD) in bone marrow (BM), assessed by next–generation flow cytometry (NGF) or next–generation sequencing (NGS), is now a central prognostic biomarker in multiple myeloma and is endorsed by the International Myeloma Working Group (IMWG) for MM patients.^1^ MRD negativity correlates with longer progression–free survival (PFS) and overall survival (OS) across treatment settings.^2^^,^^3^ Positron emission tomography/computed tomography (PET–CT) visualises focal and extramedullary disease beyond the reach of marrow sampling and is the reference functional imaging modality in MM. PET–CT negativity after therapy independently predicts improved outcomes,^4^ underscoring its potential complementarity to marrow MRD assessment.

However, accumulating evidence suggests that the two tests interrogate different biological compartments. Patchy marrow infiltration and spatial heterogeneity can yield MRD negativity despite metabolically active lesions on PET–CT,^5^ whereas diffuse microscopic disease may remain below imaging sensitivity. This compartmental sampling leads to discordant results and uncertainty about their combined prognostic implications.

Between–study comparison is further hampered by heterogeneity in MRD thresholds (eg, 10^−5^ vs 10^−6^), variable PET–CT criteria, timing misalignment, and incomplete reporting of all four joint categories (MRD^–^/PET–CT^+^, MRD^–^/PET–CT^−^, MRD^+^/PET–CT^−^, MRD^+^/PET–CT^+^). Clarifying cross–modality agreement and the prognostic significance of dual negativity is therefore essential to refine risk stratification and to harmonise reporting, particularly as response–adapted strategies become more widespread.

We therefore conducted a prespecified systematic review and meta–analysis of studies with paired MRD and PET–CT assessments to: (1) quantify cross–modality agreement, including the direction of discordance; and (2) estimate the prognostic effect of dual MRD/PET–CT negativity on PFS using random–effects models with predefined sensitivity analyses.

## Methods

### Search strategy and selection criteria

We systematically searched PubMed and Cochrane CENTRAL for studies published between Jan 1, 2015, and April 30, 2025, combining MeSH terms and free–text keywords for multiple myeloma, minimal residual disease, and PET or PET–CT. Full search strategies and the eligibility framework are provided in Supplementary Methods S1–S2. Screening followed PRISMA 2020, and we additionally hand–searched reference lists, prior reviews, and conference proceedings. The preregistered OSF protocol (DOI 10.17605/OSF.IO/3CH9E) was prospectively amended on Nov 5 2025, to extend the search window with targeted searches up to Sept 30, 2025, allowing inclusion of two late–2025 reports with paired MRD/PET–CT data (Swain et al., 2025; Talarico et al., 2025). We updated the search to Jan 31, 2026 following peer review using the same prespecified strategy. No additional eligible concordance studies were identified; however, the updated search captured a more recent published survival analysis of CASSIOPET cohort (Kraeber et al., 2025), which was incorporated for the prognostic analyses. We included peer–reviewed original studies enrolling adults with MM who underwent paired BM MRD assessment (NGF or NGS) and PET–CT after therapy and reported either: (i) joint MRD/PET–CT cross–tabulations (or sufficient data to reconstruct them), or (ii) time–to–event outcomes (PFS or OS) stratified by MRD/PET–CT category. Conference abstracts were eligible only when they provided complete extractable data. We imposed no language restrictions. Ten studies met these criteria and were synthesised quantitatively. Where multiple publications arose from the same underlying cohort (for example, CASSIOPET and its updated survival analysis by Kraeber et al., 2025), these were treated as a single analytic cohort to avoid double counting (full citations in Supplementary Methods S3). We excluded studies without paired MRD and PET–CT assessments at the same clinical landmark, studies lacking sufficient data to reconstruct joint MRD/PET–CT categories, and duplicated reports from overlapping cohorts where a more complete dataset was available.

### Data extraction

We extracted or reconstructed 2 × 2 MRD/PET–CT tables using a consistent internal ordering:

a = MRD^−^/PET–CT^+^ (imaging–only disease)

b = MRD^−^/PET–CT^−^ (dual–negative; concordant)

c = MRD^+^/PET–CT^−^ (marrow–only disease)

d = MRD^+^/PET–CT^+^ (dual–positive; concordant).

When multiple publications reported overlapping cohorts, we prioritised the report with identical MRD/PET–CT time–point pairing and the most complete 2 × 2 data. Distinct landmarks from the same trial (for example, post–induction and post–transplant) were included once per outcome. For the CASSIOPET program, concordance data were derived from the primary paired report, whereas updated survival estimates were incorporated from a later publication of the same cohort (Kraeber et al., 2025), without altering the concordance dataset.

### Concordance analysis

For each study, we extracted bone marrow MRD and PET–CT results at prespecified clinical landmarks (for example, post–induction, post–consolidation, or day +100 after autologous stem–cell transplantation). These ‘trial–defined’ landmarks formed our primary dataset of MRD/PET–CT pairs. In a prespecified sensitivity analysis (‘strict pairing’), we further restricted to patients whose MRD and PET–CT assessments were performed within ≤30 days of each other at the same landmark. Per study we calculated observed agreement, Cohen's κ (with 95% CIs), Gwet's AC1, the prevalence–adjusted bias–adjusted κ (PABAK), and McNemar's test for marginal asymmetry (details in Supplementary Methods S5). Weighted κ was not used because the joint classifications are binary (MRD positive/negative; PET–CT positive/negative); weighting is intended for ordinal categories and offers no advantage in this setting. Cohen's κ was analysed and displayed on its original scale; no transformation was applied. Directional discordance contrasted MRD^–^/PET–CT^+^ vs MRD^+^/PET–CT^−^ using the study–level log–odds (negative values indicate MRD^+^/PET–CT^−^ is more frequent). Study effects were pooled with random–effects models (REML with Hartung–Knapp adjustment). We report pooled estimates with τ^2^, I^2^, and 95% prediction intervals. Because κ is highly sensitive to imbalanced marginals and outcome prevalence (the “κ paradox”),^6^ we report observed agreement alongside κ and present AC1/PABAK as prevalence–robust comparators (Supplementary Methods S5). In practice this means that κ can remain low even when the proportion of concordant MRD/PET–CT events is moderate or high, particularly when one or two joint outcome categories dominate the contingency table. In our setting, uneven prevalence of discordant states may inflate the expected agreement by chance, thereby deflating κ despite substantial observed agreement. Thus, we do not dichotomise κ using p values; instead, we interpret point estimates and 95% CIs in the context of study–specific margins and Fréchet–Hoeffding identifiability.^7^ For studies with incomplete 2 × 2 information, we derived Fréchet–Hoeffding bounds without imputation (Supplementary Methods S6) and reported bounded (non–identified) quantities accordingly. McNemar's test was used descriptively to characterise asymmetry in discordant pairs (exact test where applicable).

### Prognostic analysis

For prognosis, the primary contrast was dual negativity (MRD^–^/PET–CT^−^) vs all other MRD/PET–CT categories combined. We abstracted or reconstructed hazard ratios (HR) and analysed log (HR) using REML random–effects models with Hartung–Knapp adjustment. Heterogeneity is summarised with τ^2^ and I^2^, and we provide 95% prediction intervals for pooled effects (Supplementary Methods S7). Given the small number of studies (k), funnel plots and Egger's tests are presented as descriptive/exploratory only (recognising low power and inflated type–I error in small k). Simulation studies examining potential biases and small–sample behaviour are described in Supplementary Methods S8.

### Risk of bias assessment

For concordance outcomes, risk of bias was appraised with QUADAS–2, using signalling questions adapted to a cross–modality agreement context (patient selection; MRD test conduct/interpretation; PET–CT conduct/interpretation; flow and timing). For prognostic outcomes, we used QUIPS (study participation, study attrition, prognostic factor measurement, outcome measurement, confounding, and statistical analysis/reporting).

Two reviewers assessed each study independently after piloting the QUADAS–2 and QUIPS forms; disagreements were resolved by discussion and, when needed, a third reviewer. Domain–level judgements were summarised using traffic–light plots and weighted bar charts. In prespecified sensitivity analyses, we excluded studies judged overall “High” risk of bias on each tool and re–estimated pooled effects, reporting k, τ^2^, and I^2^ before and after exclusions. Full risk–of–bias forms and domain guidance are provided in Supplementary Methods S9.

### Protocol registration and reproducibility

The protocol (objectives, eligibility criteria, outcomes, and statistical plan) was registered on the Open Science Framework (OSF; DOI 10.17605/OSF.IO/3CH9E) and adhered to PRISMA 2020. A single protocol amendment (Nov 5, 2025) extended the search window to capture two late–2025 reports with paired MRD/PET–CT data; analytic methods were unchanged. Analyses were performed in R version 4.5.0 (R Foundation for Statistical Computing, Vienna, Austria) using metafor for random–effects meta–analysis with Hartung–Knapp adjustment, robvis for risk–of–bias visualisation, and tidyverse for data handling (see Supplementary Methods S10–S11).

### Ethics statement

This study analysed previously published aggregate data and did not involve individual patient-level data. Therefore institutional review board approval was not required.

### Informed consent

Informed consent was not required for this systematic review and meta-analysis of previously published aggregate data.

### Role of the funding source

There was no dedicated funding source for this study. All authors had access to the underlying data. The corresponding author had final responsibility for the decision to submit for publication.

## Results

### Study characteristics

**Fig. 1:**
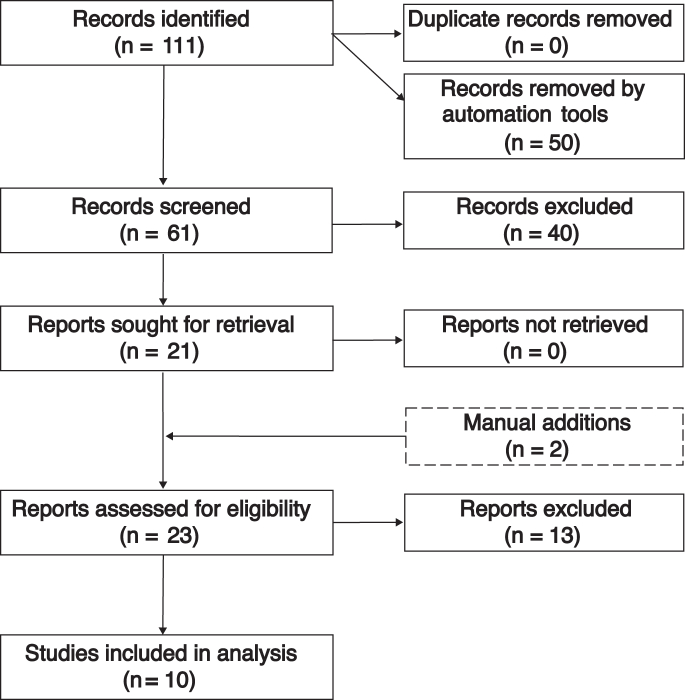
**PRISMA 2020 flow diagram of study selection**. Flow diagram showing identification, screening, eligibility assessment, and inclusion of studies evaluating paired MRD and PET–CT data. Reasons for full-text exclusion are detailed in Supplementary Table S1. MRD: minimal residual disease; PET–CT: positron emission tomography/computed tomography.

We identified 111 records (PubMed n = 69; CENTRAL/registries n = 42). Fifty records were automatically pre-excluded as non-eligible record types, leaving 61 records for title/abstract screening; 40 were excluded at this stage. We sought 21 full texts for eligibility assessment (all retrieved), assessed 23 full texts including 2 manual additions, excluded 13 with reasons, and included 10 studies in the qualitative and quantitative syntheses (Fig. 1; Supplementary Table S1). Ten studies contributed 1138 paired MRD/PET–CT assessments; five provided PFS estimates for the prognostic meta–analysis. Cohorts spanned post–induction and post–ASCT landmarks. BM MRD was assessed by NGF or NGS at sensitivities between 10^−4^ and 10^−6^. PET–CT interpretation was visual or based on Deauville criteria (Table 1). Detailed screening flow is provided in Supplementary Table S2.

**Table 1:** Study characteristics.

Study	Year	N	MRD platform	PET criteria	Simultaneous MRD and PET–CT Assessments	HR available
Alonso R et al., 2019	2019	103	Flow cytometry (10^−4^)	Italian MM imaging criteria (IMPeTUs)	Yes	Yes
CASSIOPET (Moreau et al., 2019; updated follow-up Kraeber et al., 2025)	2019	176	Flow cytometry (10^−5^)	Italian MM imaging criteria (IMPeTUs)	Yes	Yes
Zamagni E et al., 2021	2021	228	NGF (8-color, BM) (10^−5^, 10^−6^)	Deauville Criteria	Yes	No/NA
Broeckle D et al., 2022	2022	78	Flow cytometry (10^−6^)	ESMO-IMWG 2021 standard imaging criteria	Yes	No/NA
Fonseca R et al., 2023	2023	136	NGS (clonoSEQ) (10^−6^)	Binary (+/−) interpretation (radiologist read)	Yes	Yes
Mookerjee A et al., 2023	2023	131	Flow cytometry (10^−6^)	DS 2–3 = negative, DS 4 = positive	Yes	Yes
Zamagni E et al., 2023	2023	109	Flow cytometry (10^−5^)	Italian MM imaging criteria (IMPeTUs)	Yes	Yes
Hajiyianni M et al., 2024	2024	72	Flow cytometry (10^−5^)	Italian MM imaging criteria (IMPeTUs)	Yes	No/NA
Swain et al., 2025	2025	82	Flow cytometry (10^−5^)	IMWG criteria (negativity per CMR definition)	Yes	No/NA
Talarico et al., 2025	2025	23	NGS (10^−4^, 10^−5^)	Italian MM imaging criteria (IMPeTUs)	Yes	No/NA

Characteristics of the ten included cohorts with paired MRD and PET–CT assessments: study, year, number of paired assessments, MRD assay (next-generation flow cytometry or next-generation sequencing; sensitivity 10^−4^–10^−6^), PET–CT response criteria, whether MRD and PET–CT were assessed at the same time point, and availability of progression-free survival hazard ratios. MRD: minimal residual disease. NGF: next-generation flow cytometry. NGS: next-generation sequencing. PET–CT: positron emission tomography/computed tomography. HR: hazard ratio.

### Concordance between MRD and PET–CT

**Fig. 2:**
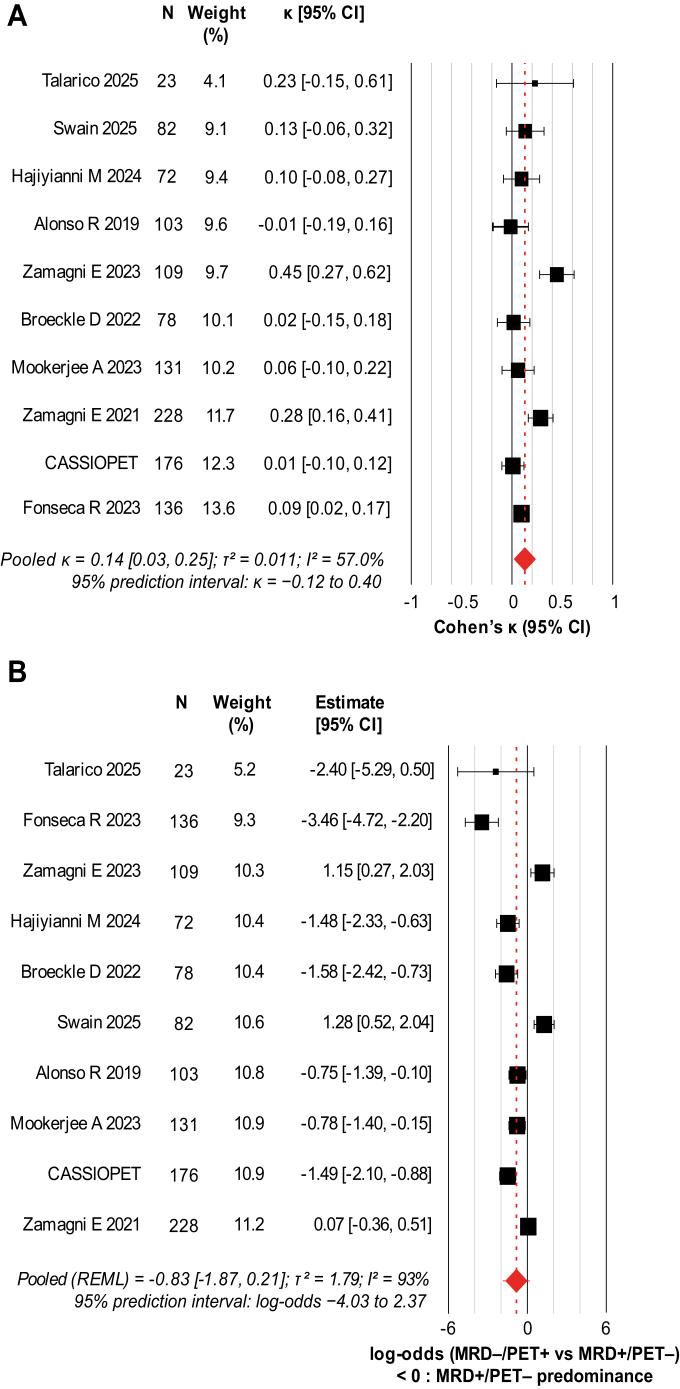
**A**. **Agreement between MRD and PET–CT across studies (Cohen's κ)**. Forest plot of study-level Cohen's κ estimates with 95% confidence intervals for agreement between bone-marrow MRD and PET–CT. κ values are shown on their original scale. Square size is proportional to inverse-variance weights from a random-effects model (restricted maximum likelihood with Hartung–Knapp adjustment). The solid vertical line indicates κ = 0 (agreement equal to chance), and the diamond denotes the pooled estimate. Between-study heterogeneity (τ^2^ and I^2^) and 95% prediction intervals are shown in the figure. MRD: minimal residual disease; PET–CT: positron emission tomography/computed tomography; CI: confidence interval. **B**. **Directional discordance between MRD and PET–CT (log–odds of MRD^–^/PET–CT^+^ vs MRD^+^/PET–CT**^–^**)**. Forest plot of study-level directional discordance, defined as the study-level log-odds comparing MRD^–^/PET–CT^+^ to MRD^+^/PET–CT^–^ classifications. A continuity correction of 0.5 was applied when a discordant cell was zero. Squares are proportional to inverse-variance weights under a random-effects model (restricted maximum likelihood with Hartung–Knapp adjustment), and horizontal bars show 95% confidence intervals. The vertical line marks log-odds = 0 (no directional imbalance), and the dashed line marks the pooled estimate. Positive values indicate imaging-dominant discordance. Between-study heterogeneity (τ^2^ and I^2^) and 95% prediction intervals are shown in the figure. MRD: minimal residual disease; PET–CT: positron emission tomography/computed tomography.

Across 1138 paired assessments pooled cohorts, the joint 2 × 2 distribution was: (a) MRD^–^/PET–CT^+^ 145 (12.7%), (b) MRD^–^/PET–CT^−^ 499 (43.8%), (c) MRD^+^/PET–CT^−^ 310 (27.2%), and (d) MRD^+^/PET–CT^+^ 184 (16.2%); observed agreement was 60.0% (Supplementary Figure S1; Supplementary Tables S3 and S4). The pooled κ was 0.14 (95% CI 0.033–0.25; I^2^ 57.0%; τ^2^ 0.011; 95% prediction interval −0.13 to 0.41; k = 10), indicating low chance–corrected agreement (Fig. 2A).

Directional discordance favoured MRD^+^/PET–CT^−^ over MRD^–^/PET–CT^+^ (Fig. 2B). The primary pooled log–odds was −0.83 (95% CI –1.87 to 0.21; τ^2^ 1.79; I^2^ 93%; k = 10). Under strict pairing (assessments within ≤30 days), the direction of discordance was unchanged (pooled log–odds −0.67, 95% CI –2.02 to 0.68; τ^2^ 2.24; I^2^ 94%; k = 8; Supplementary Figure S2). Fréchet–Hoeffding bounds showed that κ was only weakly identified in most studies, with intervals frequently crossing zero (Supplementary Table S5; Supplementary Figure S3A). Nonetheless, pooled estimates were stable across alternative τ^2^ estimators (REML, Paule–Mandel, Sidik–Jonkman, and DerSimonian–Laird; Supplementary Table S6). Simulation analyses indicated that under–reporting discordant cells biases κ toward the null, whereas complete reporting of all four MRD/PET–CT categories mitigates this distortion (Supplementary Figure S3B).

### Prognostic impact of dual negativity

**Fig. 3:**
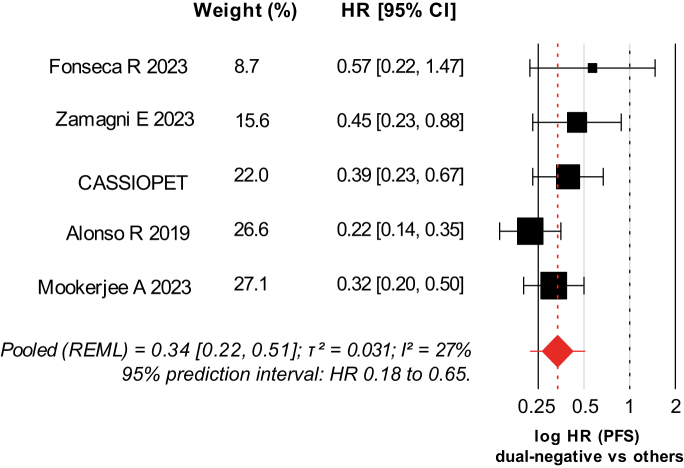
**Dual–negative MRD**^−^**/PET–CT**^−^**vs all other states: pooled hazard ratio for progression–free survival**. Random-effects meta-analysis of hazard ratios for progression-free survival comparing dual-negative MRD^–^/PET–CT^–^ status with all other MRD/PET–CT categories. Study-specific hazard ratios are displayed on a logarithmic scale as squares proportional to inverse-variance weights; horizontal bars show 95% confidence intervals. The vertical dotted line is set at HR = 1, and the diamond denotes the pooled estimate from a random-effects model. Between-study heterogeneity (τ^2^, I^2^) and the 95% prediction interval are reported in the figure. Values < 1 indicate a lower risk of progression for dual-negative MRD^−^/PET–CT^−^ status. MRD: minimal residual disease; PET–CT: positron emission tomography/computed tomography; HR: hazard ratio; CI: confidence interval.

In five studies reporting PFS by joint MRD/PET–CT status, dual negativity (MRD^–^/PET–CT^−^) was consistently associated with longer PFS than each comparator. Pooling MRD^–^/PET–CT^−^ vs all other categories yielded an HR of 0.34 (95% CI 0.22–0.51; τ^2^ 0.03; I^2^ 27%; k = 5; Fig. 3; Supplementary Table S7). Excluding studies at High risk on QUIPS gave a similar estimate (HR 0.38, 95% CI 0.28–0.51; I^2^ 0%; k = 4; Supplementary Table S8). Given the small number of studies, publication–bias assessments were considered descriptive only (Supplementary Figure S4). Leave–one–out analyses indicated no single study drove the pooled effect (Supplementary Table S8).

### Risk of bias

Domain–level QUADAS–2 (concordance) and QUIPS (prognosis) assessments are shown in Supplementary Figures S5–S6. Most studies were at low to moderate risk of bias across domains. Excluding studies judged High risk on QUADAS–2 did not materially change the pooled κ, indicating that the limited cross–modality concordance is unlikely to be explained by study quality. For prognostic outcomes, QUIPS ratings were incomplete for some cohorts, so we did not attempt formal meta–regression or stratified pooling by risk–of–bias level. Overall, however, the combination of modest κ and consistent prognostic enrichment of dual negativity was robust across prespecified sensitivity analyses, recognising the small number of studies informing the prognostic meta–analysis (Supplementary Tables S8–S9).

## Discussion

This meta–analysis demonstrates that BM MRD and PET–CT interrogate complementary rather than overlapping disease compartments. Across 1138 paired assessments, concordant results occurred in only 60% of cases, yet chance–corrected agreement was low. Despite this limited concordance, both modalities retained strong individual and joint prognostic value, supporting the interpretation that BM MRD and imaging MRD capture distinct dimensions of residual disease.

Discordance was directional. In the pooled 2 × 2 matrix, MRD^+^/PET–CT^–^ (27.2%) classifications were more frequent than MRD^–^/PET–CT^+^ (12.7%). The primary pooled log–odds was −0.83 (95% CI –1.87 to 0.21; I^2^ 93%), with a similar estimate observed in the prespecified strict pairing analysis (−0.67, 95% CI –2.02 to 0.68; I^2^ 94%). This pattern is biologically plausible, as highly sensitive NGF and NGS can detect diffuse molecular disease below the spatial resolution of PET–CT.^8^ The direction and interpretation of discordance remained unchanged in prespecified sensitivity analyses, including strict time-pairing, supporting the robustness of this finding.

Although observed agreement was about 60%, κ was lower because the four MRD/PET–CT categories were not evenly distributed across the cohorts. When discordant states are unevenly distributed, the expected agreement by chance increases, which can reduce κ despite substantial raw concordance. We therefore interpreted κ alongside observed agreement and prevalence-robust complementary measures rather than in isolation and focused on whether joint MRD/PET–CT status, particularly dual negativity, showed consistency with PFS.

In our analysis dual negativity was consistently associated with improved PFS. Pooling MRD^–^/PET–CT^−^ vs all other categories yielded an HR for PFS of 0.34 (95% CI 0.22–0.51; τ^2^ 0.03; I^2^ 27%, k = 5), with similar estimates in sensitivity analysis. Excluding studies at overall high risk of bias and leave–one–out analyses did not materially alter the pooled effect (HR 0.38, 95% CI 0.28–0.51; I^2^ 0%; k = 4), suggesting that concordant negativity is associated with a reproducibly low–risk profile, whereas discordant patterns reflect biologically heterogeneous residual disease states that are not prognostically equivalent.

Our findings align with emerging trial data. In CAR–T treated populations, early dual negativity has been associated with durable responses, whereas dual positivity confers poorer prognosis.^9^ In IMROZ and MASTER,^10^^,^^11^ achieving dual negativity translated into prolonged PFS, which in the latter study supported MRD–guided treatment discontinuation strategies. Large phase 3 trials such as GMMG–HD7 have also reported low concordance between MRD and PET–CT, reinforcing the importance of pre–specified joint reporting.^12^ Although current guidelines encourage harmonisation of MRD techniques and the integration of functional imaging,^13^ routine reporting of outcomes by combined MRD/PET–CT status remains uncommon. This gap underscores the relevance of our analysis: dual negativity is increasingly associated with favorable outcomes, yet systematic evaluation of concordance and discordance is rarely incorporated in trial designs. Quantitative integration of MRD and PET–CT therefore supports risk–adapted trial designs based on joint categories rather than prioritising a single modality. Similar patterns reported for whole-body MRI –often discordant with marrow MRD yet identifying patients with excellent outcomes –further support the rationale for multimodal MRD assessment in future studies.^14^^,^^15^

These findings have practical implications for response interpretation. While dual MRD/PET–CT negativity is consistently associated with favourable outcomes, discordant results should not be considered equivalent to deep remission based on a single modality. Moreover, implementation should be pragmatic: PET–CT access and capacity vary across healthcare systems, and universal dual-modality assessment for all patients may not be feasible. Our results provide support for evaluating risk-stratified approaches to joint assessment and prospective, response-adapted strategies. By explicitly separating concordance, directional discordance, and prognostic modelling within a four-cell joint classification, this approach may assist future studies in reporting multimodal MRD results in a reproducible manner.

Several limitations warrant consideration. First, the analysis relies on aggregated study–level data, precluding adjustment for important prognostic factors and limiting exploration of effect modification. Second, MRD sensitivity thresholds (10^−5^ vs 10^−6^) and technique (NGS vs NGF), PET–CT interpretation criteria (visual vs IMPeTUs), treatment regimens, and timing of assessments varied across cohorts, contributing to heterogeneity. Third, the limited number of studies providing survival data restricted power for subgroup or meta–regression analyses. Fourth, most included cohorts were treated in pre-quadruplet or early immunotherapy eras. Although the complementary nature of marrow-based MRD and imaging assessment is unlikely to be therapy-specific, the prevalence and magnitude of prognostic effects may differ in contemporary treatment settings; extrapolation to patients treated exclusively with modern immunotherapy-based regimens should be done cautiously. Nevertheless, multiple prespecified sensitivity analyses supported the robustness of our main conclusions: low chance–corrected concordance, directional discordance favouring MRD^+^/PET–CT^–^, and a consistent prognostic advantage associated with dual negativity.

In summary, MRD and PET–CT function as complementary rather than redundant, prognostic tools in multiple myeloma. Their combined negativity consistently identifies a biologically favourable subgroup, whereas discordant states capture distinct high–risk phenotypes that would be missed by single–modality assessment. Future myeloma trials should prespecify and report all four MRD/PET–CT categories, consider concordance and discordance as explicit secondary endpoints, and prospectively evaluate response–adapted strategies informed by joint MRD/PET–CT status.

## Contributors

M Mendez–Lopez conceived and designed the study, developed the analytic strategy, performed the statistical analysis and wrote the manuscript (writing–original draft; writing–review & editing). C Driessen contributed to data curation, interpretation, clinical contextualisation and writing (writing–review & editing). M Talarico contributed to data curation, interpretation and writing (writing–review & editing). All authors had access to and verified the underlying data, had final responsibility for the decision to submit for publication, and approved the final manuscript.

## Data sharing statement

De–identified extracted datasets, study–level reconstruction tables, and R scripts used for this meta–analysis are publicly available on GitHub (https://github.com/MaxMendezL/MRD–PET–CT–Concordance–in–Multiple–Myeloma) and archived on the Open Science Framework (OSF; DOI: 10.17605/OSF.IO/3CH9E).

## Declaration of interests

We declare no competing interests.
