# Supplementary material for: Concordance and prognostic value of bone–marrow MRD and PET–CT in multiple myeloma: a systematic review and meta–analysis
Source: eClinicalMedicine. 2026 Apr 6;94:103868. doi: 10.1016/j.eclinm.2026.103868 (PMC13090622; doi:10.1016/j.eclinm.2026.103868)
Supplement: Supplementary Methods, Figures and Tables [file mmc1.pdf]

Supplementary Appendix

Minimal concordance but complementary prognostic value of bone-marrow MRD and PET-CT in multiple myeloma: a systematic review and meta-analysis

Max Mendez-Lopez, Marco Talarico, Christoph Driessen

This Supplementary Appendix provides additional methodological detail, extended results, and risk-of-bias materials for the systematic review and meta-analysis reported in the main manuscript.

Contents

Supplementary Methods
S1. Search strategy syntax
S2. Eligibility framework (PICO)
S3. Included studies: full citations
S4. Reconstruction of 2×2 tables
S5. Agreement definitions and calculations (agreement, κ, AC1, PABAK; McNemar exact)
S6. Fréchet–Hoeffding bounds derivations
S7. Statistical analysis details (HK-REML; τ^2 estimators; prediction intervals; influence diagnostics)
S8. Simulation of under-reporting (impact of missing discordant cells on κ)
S9. Risk-of-bias forms and domain guidance (QUADAS-2; QUIPS)
S10. Statistical software and versions
S11. Data and code availability

Supplementary Figures
Figure S1. Pooled 2×2 totals.
Figure S2. Strict pairing analyses ($\Delta \leq 30$ days): agreement, κ, and directional log-odds
Figure S3. A. Fréchet–Hoeffding bounds and B. Simulation: effect of under-reporting discordant cells on κ (10 000 replicates)
Figure S4. Small-study effects (funnel plots; descriptive only)
Figure S5. QUADAS-2 traffic-light and weighted bar charts
Figure S6. QUIPS traffic-light and weighted bar charts

Supplementary Tables
Table S1. PRISMA counts (identified, screened, sought for retrieval, full-text assessed, excluded with reasons, included)
Table S2. Complete screening log (study-level)
Table S3. Per-study concordance metrics (agreement, κ [95% CI], AC1, PABAK, McNemar p, directional log-odds)
Table S4. Study-level 2×2 counts and percentages (final analytic set)
Table S5. Fréchet–Hoeffding bounds & identifiability class per study
Table S6. Model-wise τ^2 estimators (REML, Paule–Mandel, Sidik–Jonkman, and DerSimonian–Laird)
Table S7. PFS: per-study HRs, model inputs, and robustness (leave-one-out; strict pairing; high-risk excluded)
Table S8. Sensitivity analyses summary: leave-one-out, strict pairing and high-risk exclusion
Table S9. QUADAS-2 and QUIPS domain matrices (per study)
Table S10. Summary of sensitivity analyses (strict pairing, High-risk excluded)

Supplementary Methods

S1. Search strategy

PubMed: ('Multiple Myeloma'[MeSH Terms] OR 'multiple myeloma'[All Fields]) AND ('Neoplasm, Residual'[MeSH Terms] OR 'residual disease'[All Fields] OR MRD[All Fields]) AND ('Positron-Emission Tomography'[MeSH Terms] OR 'PET'[All Fields] OR 'PET-CT'[All Fields] OR 'FDG-PET'[All Fields]) Filters: 2015/01/01–2025/04/30; Humans; no language restriction. CENTRAL: similar query using MeSH and keyword equivalence. The PubMed search was updated using the same prespecified strategy to include studies up to January 2026, all analytic steps remained unchanged. Newly identified records were screened de novo. Records retrieved in earlier searches but not re-identified in the update were retained in the screening log to preserve a complete audit trail.

S2. Eligibility framework (PICO)

Element	Definition
Population	Adults with multiple myeloma after induction or post-ASCT at prespecified landmarks.
Intervention/Comparator/Index test	Bone-marrow MRD (NGF/NGS; 10^{-4}–10^{-6}) and PET-CT (visual/IMPeTUs), assessed at prespecified clinical landmarks; a prespecified 'strict pairing' sensitivity restricted to MRD and PET-CT performed within $\Delta \leq 30$ days at the same landmark.
Outcome	2×2 joint categories; agreement metrics (agreement, κ, AC1, PABAK; McNemar); directional discordance (log-odds of MRD–/PET-CT+ vs MRD+/PET-CT–); PFS HR for dual negativity vs others.
Study design	Peer-reviewed original studies; abstracts excluded unless full data available.

S3. Studies included in the Concordance and Meta-Analysis (10 concordance cohorts; 11 publications including an updated survival report) [1-11]

1. Alonso R, Cedena MT, Gómez-Grande A, et al. Imaging and bone marrow assessments improve minimal residual disease prediction in multiple myeloma. *Am J Hematol* 2019; **94**(8): 853–61.
2. Moreau P, Zweegman S, Perrot A, et al. Evaluation of the Prognostic Value of Positron Emission Tomography-Computed Tomography (PET-CT) at Diagnosis and Follow-up in Transplant-Eligible Newly Diagnosed Multiple Myeloma (TE NDMM) Patients Treated in the Phase 3 Cassiopeia Study: Results of the Cassiopet Companion Study. *Blood* 2019; **134**(Supplement_1): 692–.
3. Fonseca R, Arribas M, Wiedmeier-Nutor JE, et al. Integrated analysis of next generation sequencing minimal residual disease (MRD) and PET scan in transplant eligible myeloma patients. *Blood Cancer Journal* 2023; **13**(1): 32.
4. Hajiyianni M, Sachpekidis C, Kopp-Schneider A, et al. MRD By Flow Cytometry and FDG-PET/CT for the Post-Induction Response Assessment in Patients with Multiple Myeloma Treated in the Phase 3 GMMG HD7 Study. *Blood* 2024; **144**(Supplement 1): 4701–.
5. Mookerjee A, Gupta R, Kumar R, Sharma A, Pandey RM, Kumar L. Dual assessment with multiparameter flow cytometry and 18F-FDG PET/CT scan provides enhanced prediction of measurable residual disease after autologous haemopoietic stem cell transplant in myeloma—a prospective study. *Bone Marrow Transplantation* 2023; **58**(9): 1045–7.

6. Swain RN, Jain A, Wadhera S, et al. Role of Composite Measurable Residual Disease Assessment with PET-CT and flow cytometry in Multiple Myeloma patients undergoing Autologous Transplant. *Blood Cell Therapy* 2025; **8**(3): 234–43.
7. Talarico M, Cattabriga A, Di Franco M, et al. OA-19 - Prospective Evaluation of Response Assessment in Newly-Diagnosed Multiple Myeloma with Combined use of 18F-FDG-PET/CT, Whole-Body Diffusion-Weighted MRI and MRD by Next-Generation Sequencing. *Clinical Lymphoma Myeloma and Leukemia* 2025; **25**: S13.
8. Zamagni E, Nanni C, Dozza L, et al. Standardization of (18)F-FDG-PET/CT According to Deauville Criteria for Metabolic Complete Response Definition in Newly Diagnosed Multiple Myeloma. *J Clin Oncol* 2021; **39**(2): 116–25.
9. Zamagni E, Oliva S, Gay F, et al. Impact of minimal residual disease standardised assessment by FDG-PET/CT in transplant-eligible patients with newly diagnosed multiple myeloma enrolled in the imaging sub-study of the FORTE trial. *EClinicalMedicine* 2023; **60**: 102017.
10. Böckle D, Tabares P, Zhou X, et al. Minimal residual disease and imaging-guided consolidation strategies in newly diagnosed and relapsed refractory multiple myeloma. *Br J Haematol* 2022; **198**(3): 515–22.
11. Kraeber-Bodéré F, Jamet B, Zweegman S, et al. Prognostic value of premaintenance FDG PET/CT response in patients with newly diagnosed myeloma from the CASSIOPEIA trial. *Blood* 2025; **146**(25): 3050–8.

* Kraeber et al, 2025 is linked to the CASSIOPET cohort and used for prognosis only*.

S4. Detailed reconstruction of 2×2 tables and Deduplication

4.1. Notation and clinical ordering:

We adopted the following clinical ordering of joint categories and cell labels (counts in lowercase; proportions in uppercase):

- a = MRD$^-$/PET-CT$^+$ (imaging-only disease)
- b = MRD$^-$/PET-CT$^-$ (dual-negative; concordant)
- c = MRD$^+$/PET-CT$^-$ (marrow-only disease)
- d = MRD$^+$/PET-CT$^+$ (dual-positive; concordant)

with $n = a + b + c + d$, and A = a/n, B = b/n, C = c/n, D = d/n.

Row/column marginals: p(MRD$^-$) = A + B, p(MRD$^+$) = C + D, p(PET-CT$^-$) = B + C, p(PET-CT$^+$) = A + D.
Observed agreement $\mathbf{P_o = (b + d)/n = B + D}$.
Chance agreement $\mathbf{P_e = p(MRD^-)\times p(PET\text{-}CT^-) + p(MRD^+)\times p(PET\text{-}CT^+)}$.
Cohen's kappa: $\boldsymbol{\kappa = (P_o - P_e) / (1 - P_e)}$.

The primary ('trial-defined') 2×2 matrix includes all MRD/PET-CT assessments reported at prespecified trial landmarks (N=1138). The 'strict pairing' matrix is restricted to patients with MRD and PET-CT performed within ≤30 days at the same landmark (N=890).

4.2. Reconstruction from incomplete reports

When complete 2×2 counts were unavailable, we reconstructed study-level tables under a deterministic rule-set:

1. Three percentages reported (sum ≈ 100%): The missing cell proportion was computed as 100% – (sum of reported); values were then rescaled to sum exactly to 100%.
2. Percentages reported but do not sum to 100%: All percentages were rescaled to 100%, then converted to counts by largest-remainder (Hamilton) rounding:
   - provisional counts = floor(percentage × n / 100);
   - allocate the remaining n − Σ floor to cells with the largest fractional parts (stable tie-break in the fixed order a, b, c, d). This guarantees $a + b + c + d = n$ and maximises fidelity to the reported proportions.
3. One cell count missing: The missing count was set to the remainder so that $a + b + c + d = n$.

4. ≥2 cell counts missing: If row/column marginals were reported, counts were obtained by proportional rebalancing to match both marginals (then integerised by largest-remainder). If no marginals were available, we applied rule (2) using reported percentages.

4.3. Tolerances and QC

After reconstruction, we back-calculated percentages and required absolute deviations from reported values to be ≤0·5 percentage points per cell (or flagged the record for manual check).

4.4. Deduplication

When multiple reports arose from the same underlying cohort, we selected a single dataset per clinical landmark to avoid double counting. For the *Cassiopeia* Study, imaging and MRD data were available from both the original trial report and the dedicated CASSIOPET substudy. We therefore treated these cohorts as one cohort and used the most complete, methodologically aligned dataset (CASSIOPET) for all concordance and prognostic analyses. For this cohort, progression-free survival data were updated using the most recent published analysis (Kraeber et al., 2025), which provides longer follow-up than earlier reports. This update affected survival estimates only and did not alter the concordance analyses, the underlying MRD/PET-CT classification or the composition of the analytic cohort.

S5. Agreement metrics and directional discordance

For each study, we formed a 2×2 table with the clinical ordering defined in Section S4:
a = MRD– / PET-CT+ (imaging-only disease)
b = MRD– / PET-CT– (dual-negative; concordant)
c = MRD+ / PET-CT– (marrow-only disease)
d = MRD+ / PET-CT+ (dual-positive; concordant)
n = *a* + *b* + *c* + *d*

5.1. Agreement and κ-type statistics

- **Observed agreement** was defined as the proportion of patients with concordant results:

$$P_o = (b + d)/n.$$

- **Cohen's κ** quantifies agreement beyond chance as

$$\kappa = \frac{P_o - P_e}{1 - P_e},$$

  where *P*e is the expected agreement if MRD and PET-CT were independent, calculated from the row and column margins. Because κ is sensitive to imbalanced marginals ("κ paradox"), we pre-specified complementary metrics.

- **Gwet's AC1** (binary case) uses an alternative chance agreement that down-weights extreme prevalences. Let $\bar{p}$denote the average positivity across MRD and PET-CT; the chance agreement for AC1 is defined in terms of $\bar{p}$, and AC1 is formed analogously to κ.

- **PABAK** (prevalence- and bias-adjusted κ) rescales the observed agreement as

$$\text{PABAK} = 2P_o - 1.$$

5.2. **Discordance and McNemar's test**

Discordant cells are *a* (MRD–/PET-CT+) and *c* (MRD+/PET-CT–). We used McNemar's exact test to assess asymmetry between these discordant categories; p values are reported descriptively.

5.3. Directional discordance for meta-analysis

To summarise whether residual disease was more often detected by PET-CT or by MRD, we modelled the directional log-odds of discordance:

$$\log\ \mathrm{OR}_{\mathrm{dir}} = \log\left(\frac{a}{c}\right),$$

where values >0 indicate more MRD–/PET-CT+ than MRD+/PET-CT– pairs (imaging-dominant detection).

When *a* or *c* was zero, we applied a Haldane–Anscombe continuity correction (+0·5 to both discordant cells) and verified robustness using a study-size-scaled correction in sensitivity analyses.

5.4. Uncertainty and pooling

We report 95% confidence intervals (CIs) for κ, AC1, and PABAK using standard large-sample (delta-method) formulas, and exact 95% CIs for Discordance log-odds where appropriate. Study-level directional log-odds and their standard errors were entered into random-effects meta-analyses (restricted maximum likelihood [REML] with Hartung–Knapp adjustment). We present pooled estimates together with τ^2, I^2, and 95% prediction intervals in the relevant figures and tables.

S6. Fréchet–Hoeffding bounds for κ

For some studies, the marginals of MRD and PET-CT were well reported but one or both discordant cells were incompletely specified. In these settings, the *exact* κ cannot be point-identified without further assumptions, but κ is constrained to lie within a range implied by the fixed margins. We therefore computed Fréchet–Hoeffding (FH) bounds for the observed agreement *P*o and mapped these to corresponding bounds for κ, holding the marginal distributions of MRD and PET-CT fixed. This yields an interval $[\kappa_{\min}, \kappa_{\max}]$ of all κ values that are compatible with the published margins, without imputing any discordant counts.

For interpretation we classified each study as:

- Identifiable positive: FH interval entirely above 0.
- Identifiable negative: FH interval entirely below 0.
- Non-identified: FH interval wide or crossing 0.

These classes are reported in Supplementary Table S5 and referenced in the main text when discussing the identifiability of κ. FH bounds are used as *identification sets*, not as point estimators.

S7. Sensitivity analyses and alternative τ² estimators

7.1. Random-effects variance estimators and model variants

The primary meta-analyses used random-effects models with REML estimation of τ^2 and Hartung–Knapp adjustments for inference. We repeated all pooled estimates under alternative specifications:

- Between-study variance (τ^2) estimators: Paule–Mandel (PM), Sidik–Jonkman (SJ), and DerSimonian–Laird (DL).
- Fixed-effect models (inverse-variance) as a benchmarking sensitivity.
- Prediction intervals (PI): reported using the same τ^2 as the corresponding model.

Across concordance (κ) and prognostic (HR) outcomes, pooled point estimates and heterogeneity summaries (τ^2, I^2) were stable to the choice of τ^2 estimator; where differences arose, they were within expected sampling variability and did not alter interpretation. We report model-wise summaries in the corresponding figure captions and Supplementary Tables**.**

7.2. Pairing, landmark, and method sensitivities

We re-estimated pooled effects in the following subsets to probe clinical/technical sources of heterogeneity:

- Strict time-pairing ($\Delta \leq 30$ days at the same clinical landmark).
- Risk of bias: exclusion of studies at High risk (QUADAS-2 for concordance; QUIPS for prognosis).

Effect directions were unchanged across all sensitivities; magnitude and heterogeneity varied modestly as expected with *k*and setting, without changing conclusions.

7.3. Continuity corrections for directional discordance

For study-level directional discordance (log-odds $\theta = \log{(a/c)}$), we applied a Haldane–Anscombe add-on (+0·5) to *a* and/or *c* when zeros occurred. In sensitivity analyses we used an study-size–scaled correction. Pooled log-odds estimates differed by <0·02 in absolute value and heterogeneity was essentially unchanged; inference and interpretation were unaffected. *(Note: continuity choices do not affect κ; they pertain only to the log-odds meta-analysis).*

7.4. Influence diagnostics and robustness

We conducted leave-one-out analyses and inspected influence statistics. No single study materially changed pooled κ, pooled directional log-odds, or the dual-negativity HR; all leave-one-out estimates remained within the primary 95% Cis (see Supplementary Table S8).

7.5. Small-study effects (descriptive)

Given the small number of prognostic studies (k = 5), funnel plots and Egger's tests are presented descriptively; no inferential claims are made.

S8. <u>Simulation of under-reporting bias</u>

We simulated the impact of incomplete 2×2 reporting (for example, "either-positive" formats that omit one or both discordant cells) on Cohen's κ and compared this with Fréchet–Hoeffding (FH) bounds. The simulation is illustrative and complements the empirical FH analyses (Figure S3A).

8.1. Data-generating mechanism

- We drew marginal MRD and PET-CT prevalences by sampling with replacement from the study-level margins observed in the included cohorts (Supplementary Table S3).
- For each of 10 000 replicates we then generated a complete 2×2 table with total sample size *n* sampled from the empirical distribution of study sizes. Cell probabilities were centred on the empirical means so that the distribution of κ resembled that of the observed studies.
- The resulting "true" tables provided the gold-standard κ values.

8.2. Missingness scenarios

From each true table we constructed three types of incomplete report:

1. Omission of $MRD^{-}/PET\text{-}CT^{+}$ (cell *a*).
2. Omission of $MRD^{+}/PET\text{-}CT^{-}$ (cell *c*).
3. Either-positive summary, in which discordant cells are combined or incompletely reported.

In scenarios 1. and 2., we mimicked common practice by reconstructing the missing discordant cell via proportional scaling from the remaining cells, ensuring totals matched. We then recomputed a naïve κ from the reconstructed table.

8.3. Estimators compared

- Naïve κ from incomplete tables after proportional back-fill.
- FH κ interval, computed from the reported margins without any imputation.
- True κ from the complete 2×2 table.

For κ point estimates we summarised bias, root mean squared error (RMSE), and 95% CI coverage**.** For FH intervals we reported the proportion of intervals containing the true κ and the median interval width, stratified by marginal imbalance and prevalence.

8.4. Interpretation (Figure S3B)
Across scenarios, naïve κ derived from incomplete 2×2 tables was systematically biased toward 0, particularly when discordance was common and margins were imbalanced—closely matching the empirical setting of this review. FH intervals were typically wide but well-calibrated in that they contained the true κ at high frequency. Full simulation code (including random seeds) is available in the public repository.

S9. Risk-of-bias (QUADAS-2 and QUIPS)

9.1. Tools and scope

- **Concordance outcomes: QUADAS-2**, adapted to cross-modality agreement (bone-marrow MRD vs PET-CT) at defined clinical landmarks.
- **Prognostic outcomes: QUIPS** for studies reporting PFS by joint MRD/PET-CT status.

Two reviewers performed independent assessments after piloting; disagreements were resolved by consensus, with arbitration by a third reviewer if needed. Per-domain judgements are visualised in Supplementary Figures S5–S6 as traffic-light and weighted bar charts.

9.2. QUADAS-2 (concordance)

Domains and signalling questions (adapted)

- **Patient selection:**
  - Consecutive or random series?
  - Avoided case-control or enriched designs?
  - Prespecified inclusion/exclusion, with no post-hoc selection by MRD/PET-CT result?
    Risk of bias: *High* if convenience sampling or exclusions tied to either test result; *Unclear* if sampling frame not reported.
    Applicability: *High concern* if population not representative of post-induction or post-ASCT adults (e.g., solitary plasmacytoma).
- **Index test (MRD: NGF/NGS):**
  - Assay described (NGF/NGS), threshold stated (10^{-4}–10^{-6}), IMWG-concordant?
  - Operator blinding to PET-CT?
  - Valid internal QC/LoD and quality criteria (cell events, sequencing depth)?
    Risk of bias: *High* if threshold or QC absent, or unblinded with plausible influence.
    Applicability: *High concern* if assay not standard (e.g., legacy flow with sub-IMWG sensitivity).
- **Reference test (PET-CT):**
  - Interpretation method described (visual vs Deauville/IMPeTUs)?
  - Reader blinding to MRD?
  - Acquisition adequate (whole-body coverage, FDG prep, timing post-therapy)?
    Risk of bias: *High* if unblinded reads or non-standard criteria likely alter positivity.
    Applicability: *High concern* if protocol differs markedly from contemporary PET-CT practice.
- **Flow and timing:**
  - Same clinical landmark for both tests (e.g., day +100 post-ASCT)?
  - **Pairing interval $\Delta \leq 30$ days** met?
  - All eligible patients included in the 2×2 (no partial verification)?
    Risk of bias: *High* if $\Delta > 30$ days or landmark mismatch without justification, or if exclusions depend on test results.
    Applicability: *High concern* if timing/landmark mismatched to clinical question.

Prespecified use in analysis: Studies rated High risk overall (any critical domain at High) were excluded in sensitivity analyses; we report k, τ^2, I^2, and pooled estimates before/after exclusions.

9.3. QUIPS (prognosis)

Domains and signalling questions (adapted)

- **Study participation**: Source population well described and representative of treated MM adults at the stated landmark?. High RoB if selective inclusion likely.
- **Study attrition**: Follow-up adequate and balanced across MRD/PET-CT strata; reasons for loss reported?. High RoB if differential loss to follow-up.
- **Prognostic-factor measurement (joint MRD/PET-CT)**: MRD and PET-CT defined as above; timing aligned; cut-offs prespecified; assessors blinded?. High RoB if reclassification post-hoc or unclear timing.
- **Outcome measurement (PFS/OS)**: Standard definitions; blinded adjudication or objective criteria; uniform schedule?. High RoB if outcome ascertainment differs by subgroup.
- **Confounding**: Key factors (age, ISS/R-ISS, cytogenetics, therapy class/lines, ASCT) measured and handled (adjusted analyses or stratification)?. High RoB if major confounders unmeasured/unadjusted.
- **Statistical analysis and reporting**. HRs derived appropriately (time-to-event methods, proportional hazards checked or justified); complete reporting (events, censoring, strata counts)?
  High RoB if model misspecification or selective reporting.

Prespecified use in analysis: We performed a sensitivity meta-analysis excluding High overall QUIPS studies and reported changes in pooled HR, τ^2, I^2, and k.

9.4. Handling "Unclear" and partial information

Where reporting was insufficient, we assigned Unclear rather than assuming Low risk. For overlapping reports from the same cohort, we selected the most complete, best-aligned landmark dataset to avoid double counting; RoB reflects the chosen report.

9.5. Visualisation and data availability

- **Figures S5–S6:** Per-domain traffic-light and weighted bar summaries (robvis).
- **Data:** Study-level RoB matrices (per domain and overall) are provided in Tables S9–S10 with justifications keyed to verbatim study quotes.

S10. Statistical software and versions

Analyses were performed in R version 4·5·0 (R Foundation for Statistical Computing, Vienna, Austria) on macOS/Linux. Graphics used base R and ggplot2. Meta-analyses used the *metafor* package (random-effects, REML, Hartung–Knapp); exact tests used *exact2x2*; risk-of-bias plots were generated using *robvis*; data handling used *tidyverse*.

S11. Data and code availability

All materials required to reproduce the results are available on the **Open Science Framework** (OSF) under DOI **10.17605/OSF.IO/3CH9E** and Github under the repository:
https://github.com/MaxMendezL/MRD-PET-CT-Concordance-in-Multiple-Myeloma

Supplementary Figures

Figure S1. Pooled MRD × PET-CT concordance matrices at primary and strict time-points

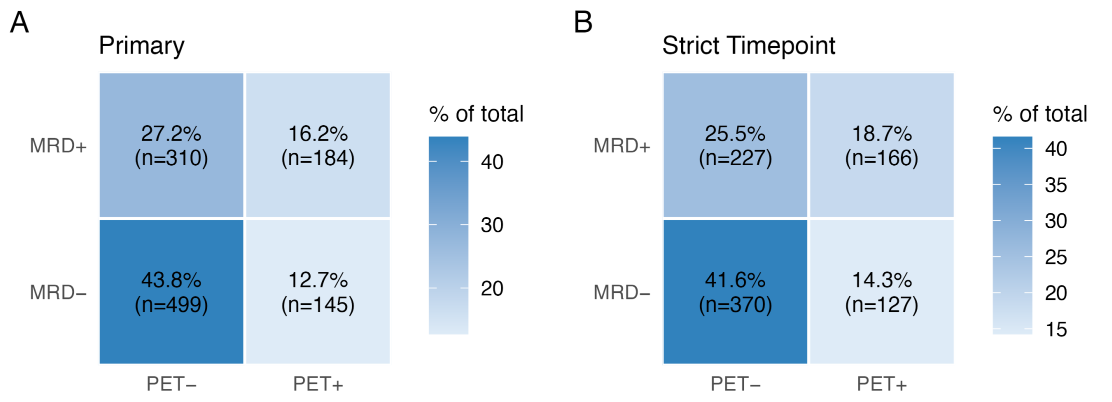

Heatmaps showing pooled 2×2 MRD × PET-CT counts at (A) the primary trial-defined MRD/PET-CT landmarks and (B) under strict time-pairing ($\Delta \leq 30$ days at the same clinical landmark). Each cell displays the percentage of the total analytic sample and the corresponding count (*n*). Strict-pairing matrices are restricted to patients with MRD and PET-CT performed within $\Delta \leq 30$ days at the same clinical landmark.

Figure S2. Directional discordance under strict pairing: study-level log-odds and pooled effect

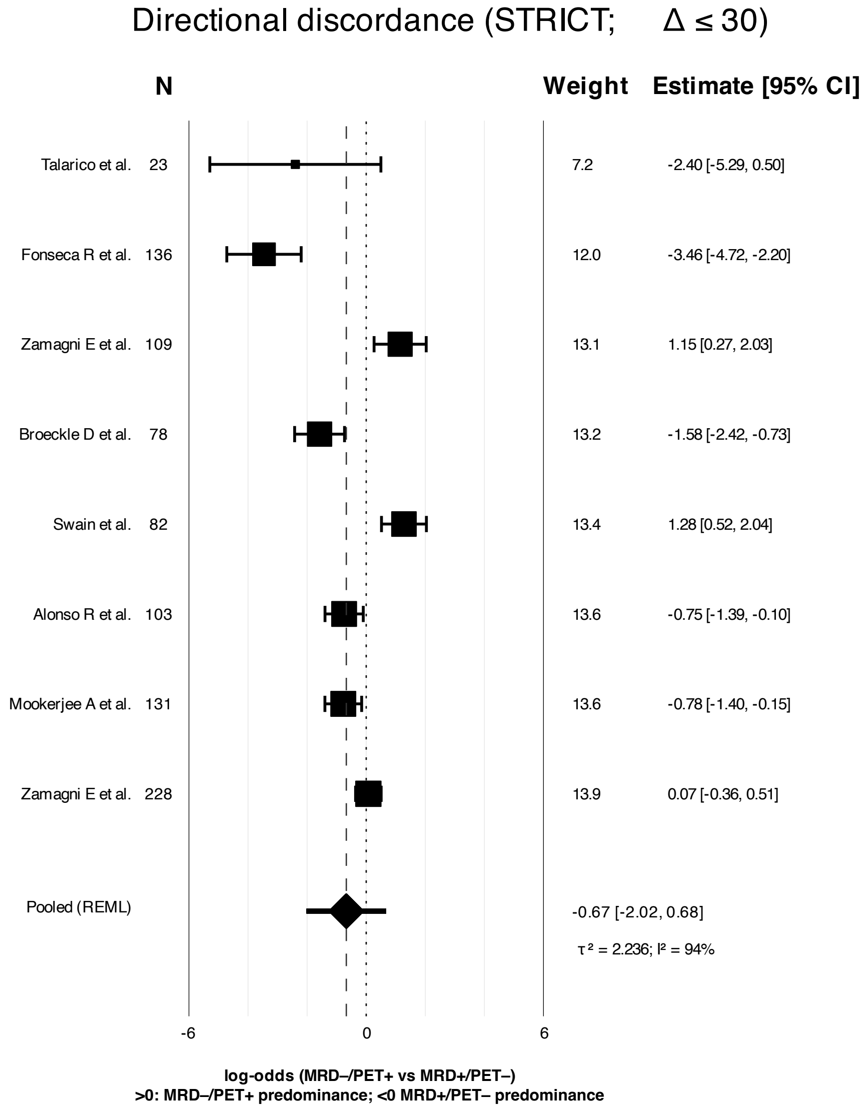

Forest plot of study-level log-odds comparing MRD$^-$/PET-CT$^+$ versus MRD$^+$/PET-CT$^-$ among patients with strictly paired assessments (Δ ≤ 30 days). Squares are sized according to inverse-variance weights from a random-effects model (REML), and horizontal bars represent 95% confidence intervals. A Haldane–Anscombe correction (+0.5) was applied if either discordant cell was zero. The dotted vertical line indicates log-odds = 0 (no directional imbalance), and the dashed line marks the pooled estimate. The random-effects model uses REML with Knapp–Hartung adjustment; τ^2, I^2, and the 95% prediction interval are reported in the figure caption. Positive log-odds values indicate studies in which MRD$^-$/PET-CT$^+$ discordance was more common than MRD$^+$/PET-CT$^-$ ("imaging-dominant" discordance)

Figure S3. κ identification intervals (Fréchet–Hoeffding bounds) and simulation of under-reporting bias

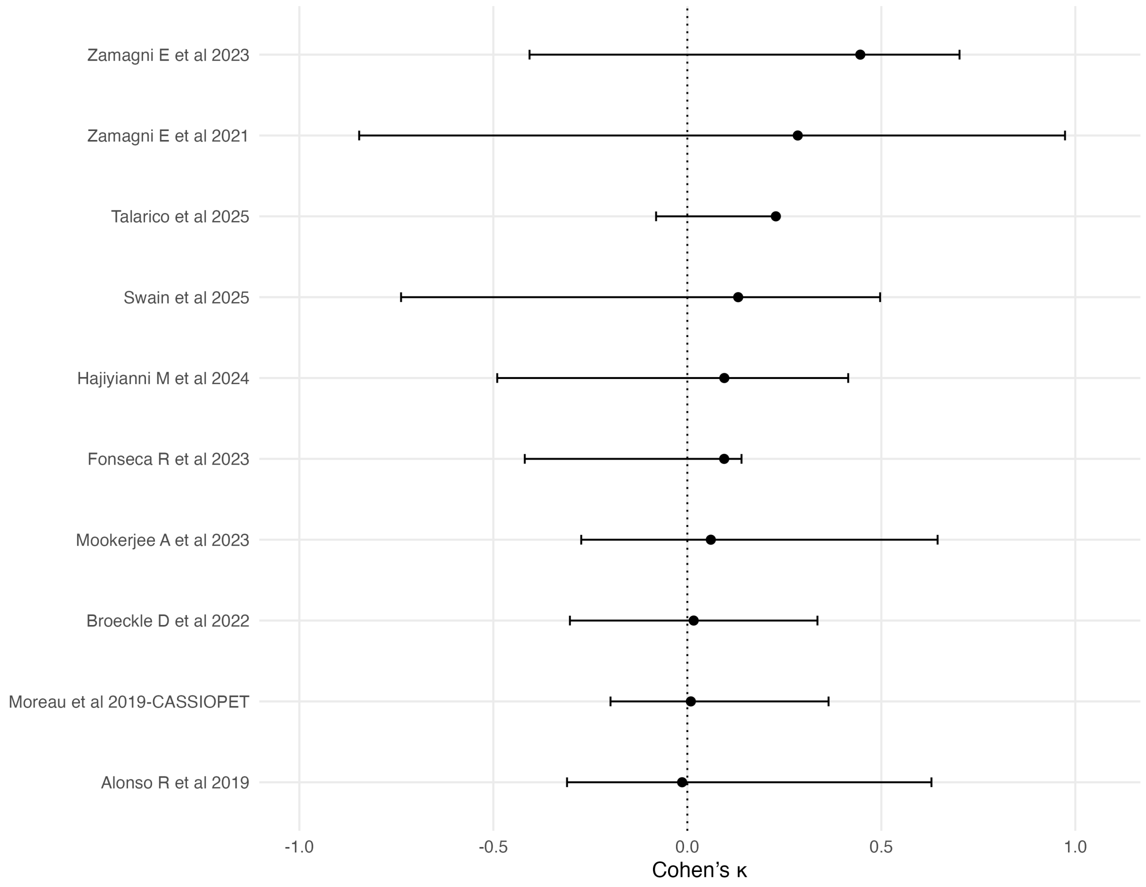

(A) Fréchet–Hoeffding (FH) identification intervals for κ by study. For each study, the FH interval $[\kappa_{\min}, \kappa_{\max}]$ is shown as implied by the reported MRD and PET-CT margins, without imputing any discordant cells. Points represent the conventional κ estimate based on the reconstructed 2×2 table (where available). Studies are classified as "identifiable positive" (interval entirely > 0), "identifiable negative" (interval entirely <0), or "non-identified" (interval wide or crossing 0), as reported in Supplementary Table S5

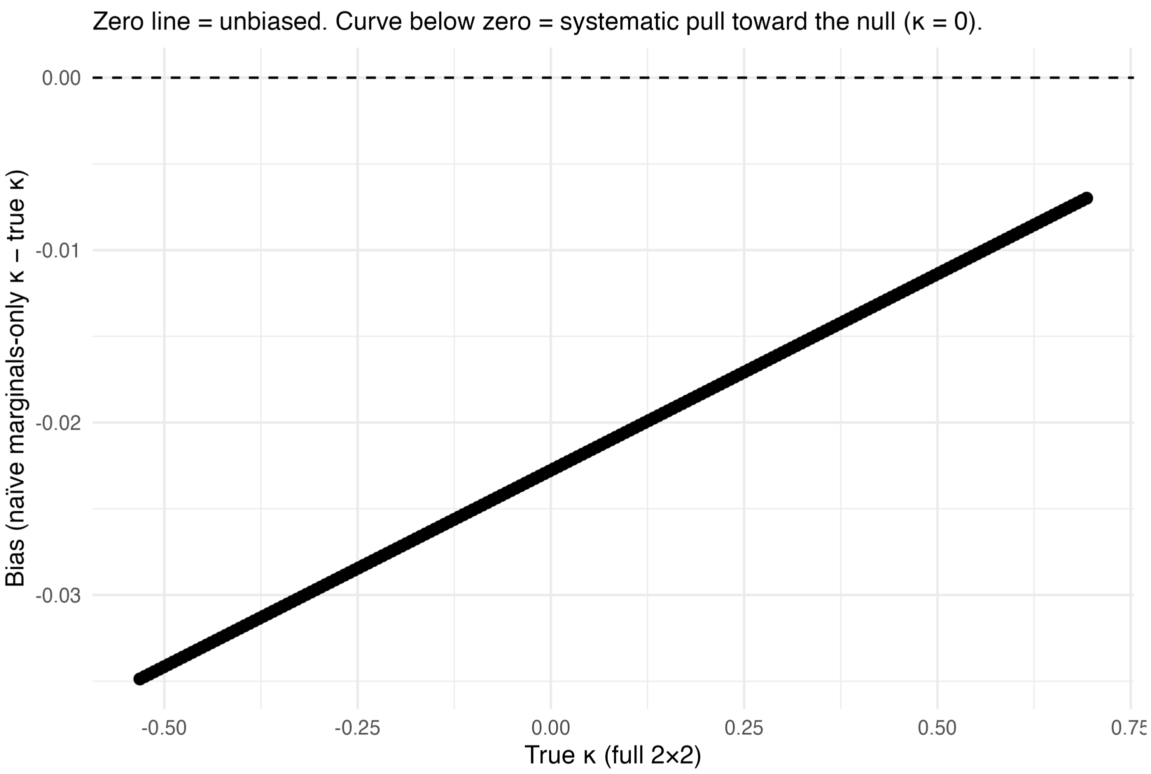

(B) Bias of naïve "marginals-only" κ. Using pooled MRD and PET-CT marginals, we sample feasible 2×2 tables and compute the true κ. We then mimic "either-positive" reporting by retaining only the total number of discordant results and naïvely splitting them equally between MRD−/PET-CT+ and MRD+/PET-CT− before recomputing κ. The figure shows bias (naïve κ minus true κ) as a function of true κ. Values below zero indicate systematic pull toward the null (κ = 0), leading to underestimation of concordance when κ > 0 and masking of negative concordance when κ < 0.

Figure S4. Small-study effects in prognostic meta-analysis (descriptive only)

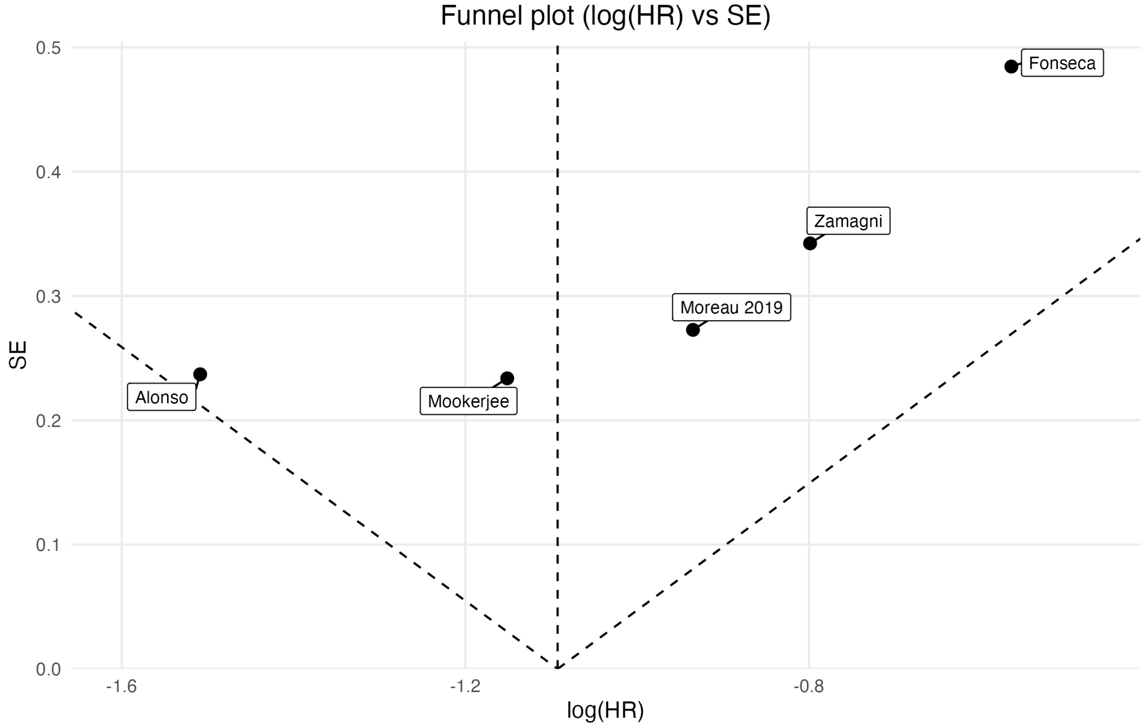

Funnel plots of log(hazard ratio) versus standard error for the progression-free survival (PFS) meta-analysis (dual-negative vs others). The vertical line indicates the pooled log(hazard ratio) from the primary random-effects model (REML with Hartung–Knapp adjustment). Pseudo 95% confidence limits for a fixed-effect model are shown for reference. Egger's regression test is provided descriptively; no inferential claims are made given the small number of studies (k = 5). HR = hazard ratio; SE = standard error.

Figure S5. QUADAS-2 risk-of-bias assessments for concordance outcomes

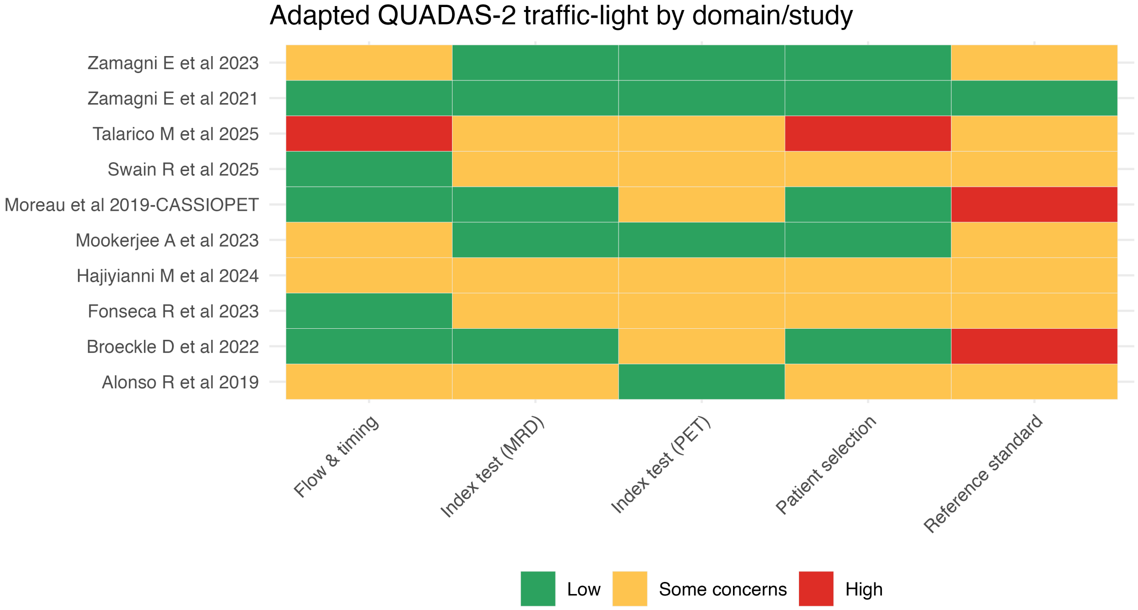

Traffic-light plots and weighted bar charts summarising QUADAS-2 domain-level and overall risk-of-bias judgements for studies contributing to the MRD/PET-CT concordance analyses. These assessments informed prespecified sensitivity analyses in which studies at overall high risk of bias were excluded from the pooled κ and directional discordance models.

Figure S6. QUIPS risk-of-bias assessments for prognostic outcomes

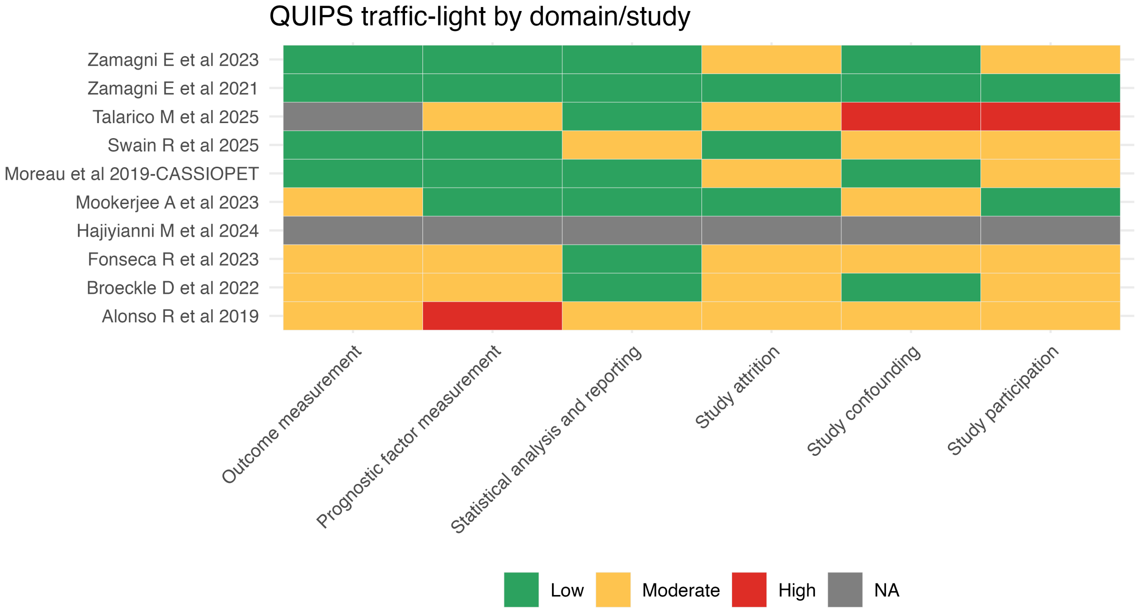

Traffic-light plots and weighted bar charts summarising QUIPS domain-level and overall risk-of-bias judgements for studies contributing to the PFS meta-analysis by joint MRD/PET-CT status. These assessments informed a prespecified sensitivity analysis excluding studies rated overall high risk on QUIPS, as reported in Supplementary Table S8.

Supplementary Tables

Supplementary Table S1. PRISMA 2020 flow: records and studies at each screening stage

Stage	Count	Explanation
Identified	111	Pubmed (n = 69); CENTRAL/registries (n = 42)
Pre-excluded non-eligible record types	50	Registration records without results, conference abstracts lacking data, articles automatically filtered (review, guidelines, editorials)
Screened (Title/Abstract)	61	All remaining original research articles
Excluded after screening	40	Records were excluded at the title/abstract stage because they clearly did not meet eligibility criteria:<br>• Review, guideline, consensus, or editorial articles without original patient data (n = 26)<br>• Case reports or small case series (n = 4)<br>• Technical/methodology or radiomics papers without MRD or survival data (n = 4)<br>• Wrong disease/population (solitary plasmacytoma or precursor states) (n = 1)<br>• No MRD assessment reported (n = 2)<br>• No PET-CT assessment relevant to the review question (n = 2)<br>• Outside prespecified timeframe (n = 1)
Sought	21	Required for full-text assessment
Not Retrieved	0	All full text were retrievable
Manual additions	2	Key studies not captured by database search
Full Text assessed	23	21 retrieved from the search plus 2 manual additions
Excluded after full text review	13	• Trial registration or ongoing study with no published result at the time of assessment (n = 4)<br>• Duplicate publication/cohort (full-text assessment) of an already included study (n = 3)<br>• Insufficient Data for Concordance or Prognostic analysis (n = 4)<br>• Wrong disease (Smoldering MM) (n = 1)<br>• Outside prespecified timeframe (n = 1)
Included	10	Included in final qualitative synthesis

Numerical counts corresponding to the PRISMA flow diagram (Figure 1).

Supplementary Table S2. Complete screening log (study-level)

Study-level log of screening decisions and reasons for exclusion.

A) Pubmed Log-Search

PMID	Title	Journal	Year	Abstract Analysis (Inclusion, Exclusion, Maybe)	Reason	Full Text Needed	Full Text Review	Included
41523083	Daratumumab-based quadruplet for patients with extramedullary multiple myeloma: Results from the Phase II prospective EMN19 study.	Hemasphere	2026	Maybe	Original prospective MM cohort with PET/CT + marrow MRD + outcomes, but abstract lacks joint PET/MRD strata (2×2) for concordance	YES	Imaging was repeated per response landmarks until CMR, MRD was assessed at ≥CR or earlier per practice; no single synchronized timepoint 2×2 is given. The paper presents response proportions and KM differences, not the joint 4-cell table at one visit	NO
41510164	Precision radiolabeled B-cell maturation nanobody for targeted PET imaging and radioligand therapy of disseminated multiple myeloma.	Theranostics	2026	Excluded	Preclinical/animal theranostic development; no human MM cohort, no clinical outcomes	NO	Excluded at abstract step	NO
41257443	Analysis of discordant results in multi-technique platform-based MRD detection in multiple myeloma and the clinical decision-making dilemma.	Leuk Lymphoma	2026	Excluded	Review article or guideline, no original patient data	NO	Excluded at abstract step	NO
41216713	Treatment response assessment using whole body diffusion weighted-magnetic resonance imaging in myeloma: a retrospective cohort study.	Leuk Lymphoma	2026	Excluded	WB DW-MRI response assessment (not PET/CT-based); not eligible for PET/CT–MRD concordance/prognosis framework	NO	Excluded at abstract step	NO
41032077	Validation of novel low-dose CT methods for quantifying bone marrow in the appendicular skeleton of patients with multiple myeloma: initial results from the [(18)F]FDG	Eur J Nucl Med Mol Imaging	2026	Excluded	PET/CT sub-study focused on CT quantification/IMPeTUs correlations; no extractable PET/CT–marrow MRD joint strata or survival outcomes for this review	NO	Excluded at abstract step	NO

	PET/CT sub-study of the Phase 3 GMMG-HD7 Trial.							
41314938	Progression-Free and Overall Survival After KRd Consolidation in FDG PET/CT Positive Patients After ASCT: A Phase II Study (CONPET).	Clin Lymphoma Myeloma Leuk	2025	Included	Clinical Phase II study with PET/CT status + marrow MRD and survival endpoints; likely extractable prognostic comparisons	YES	Included	NO
41239440	B-cell maturation antigen targeted PET/CT imaging in multiple myeloma: a first-in-human study.	J Hematol Oncol	2025	Maybe	Moved to 'Maybe'. First-in-human BCMA PET/CT; MRD mentioned but unclear whether marrow MRD testing/joint strata are reported	YES	Excluded at abstract step	NO
41038751	Treatment Accessibility, Availability, and Healthcare Costs for Multiple Myeloma in South Asian Countries.	Clin Lymphoma Myeloma Leuk	2025	Excluded	Health-systems/cost survey; not PET/CT–MRD concordance or prognostic outcomes	NO	Excluded at abstract step	NO
40991848	Prognostic value of premaintenance FDG PET/CT response in patients with newly diagnosed myeloma from the CASSIOPEIA trial.	Blood	2025	Included	CASSIOPET/CASSIOPEIA companion study: PET/CT response + marrow MRD with PFS/OS; key prognostic dataset	YES	Included	YES
40927280	Role of Composite Measurable Residual Disease Assessment with PET-CT and flow cytometry in Multiple Myeloma patients undergoing Autologous Transplant.	Blood Cell Ther	2025	Included	Original cohort using composite MRD (PET-CT + marrow flow) with prognostic endpoints (TTNT/OS)	YES	Included	YES
40875592	Fifteen years of use of functional imaging in multiple myeloma: where we started and where we are going.	Blood Adv	2025	Excluded	Review article or guideline, no original patient data	NO	Excluded at abstract step	NO
40757112	Beyond the bone marrow: a review of therapeutic approaches for	J Med Life	2025	Excluded	Narrative review; no original cohort / no extractable outcomes	NO	Excluded at abstract step	NO

	extramedullary disease in multiple myeloma and the significance of MRD assessment.							
40590883	Prognostic impact of focal lesion location and persistence in multiple myeloma: insights from serial PET/DWI imaging.	Blood Adv	2025	Maybe	Serial PET/DWI imaging cohort; MRD mentioned but unclear if same-timepoint PET + marrow MRD joint strata are reported	YES	Imaging combined PET+DWI; no PET-only same-timepoint MRD×PET 2×2; no dual-neg HR	NO
25993214	Multiple myeloma: is it time for biomarker-driven therapy?	Am Soc Clin Oncol Educ Book	2015	Excluded	Review article or guideline, no original patient data	NO	Excluded at abstract step	NO
35582835	Minimal residual disease and imaging-guided consolidation strategies in newly diagnosed and relapsed refractory multiple myeloma.	Br J Haematol	2022	Maybe	Moved to 'Maybe' - unclear mention of MRD and PET in abstract	YES	Included	YES
28561668	Established and Novel Prognostic Biomarkers in Multiple Myeloma.	Am Soc Clin Oncol Educ Book	2017	Excluded	Educational review — no original cohort, no MRD or PET-related survival outcomes	NO	Excluded at abstract step	NO
38458477	Early Chimeric Antigen Receptor T Cell Expansion Is Associated with Prolonged Progression-Free Survival for Patients with Relapsed/Refractory Multiple Myeloma Treated with Ide-Cel: A Retrospective Monocentric Study.	Transplant Cell Ther	2024	Maybe	Moved to 'Maybe' - unclear mention of MRD and PET in abstract	YES	not included in quantitative meta-analysis (e.g., for kappa or HRs) due to lack of full PET/MRD subgroup counts and HRs. Add to qualitative synthesis and CAR-T subgroup analyses, especially due to high-resolution timing and modern MRD/PET tools.	NO
27922862	Improved Detection of Minimal Residual Disease by 11C-Methionine PET/CT in a Young Patient With Unusual Extramedullary Presentation of Recurrent Multiple Myeloma.	Clin Nucl Med	2017	Excluded	Case Report	NO	Excluded at abstract step	NO

28368259	Role of (18)F-FDG PET/CT in the diagnosis and management of multiple myeloma and other plasma cell disorders: a consensus statement by the International Myeloma Working Group.	Lancet Oncol	2017	Excluded	Review article or guideline, no original patient data	NO	Excluded at abstract step	NO
37217164	How do we image patients with multiple myeloma and precursor states?	Br J Haematol	2023	Excluded	Case Report	NO	Excluded at abstract step	NO
28677897	Guidelines for the use of imaging in the management of patients with myeloma.	Br J Haematol	2017	Excluded	Review article or guideline, no original patient data	NO	Excluded at abstract step	NO
34474661	3-weekly daratumumab-lenalidomide/pomalidomide-dexamethasone is highly effective in relapsed and refractory multiple myeloma.	Hematology	2021	Included	Meets all inclusion criteria	YES	Included	YES
33285523	Defining and Managing High-Risk Multiple Myeloma: Current Concepts.	J Natl Compr Canc Netw	2020	Excluded	Educational review — no original cohort, no MRD or PET-related survival outcomes	NO	Excluded at abstract step	NO
29567784	Treatment to suppression of focal lesions on positron emission tomography-computed tomography is a therapeutic goal in newly diagnosed multiple myeloma.	Haematologica	2018	Maybe	Moved to 'Maybe' - unclear mention of MRD and PET in abstract	YES	No MRD Data	NO
32506429	Clinician attitudes and practices toward measurable residual disease in multiple myeloma.	Br J Haematol	2020	Excluded	Inclusion Criteria not met	NO	Excluded at abstract step	NO
39375362	Discontinuation of maintenance therapy in multiple myeloma guided by multimodal measurable residual disease negativity (MRD2STOP).	Blood Cancer J	2024	Maybe	Moved to 'Maybe' - unclear mention of MRD and PET in abstract	YES	eligibility required patients to be PET-negative and MRD-negative ($\geq 10^{-6}$) at baseline. That means there's no variation in baseline PET status and no joint PET/MRD strata	NO

32771227	Defining the undetectable: The current landscape of minimal residual disease assessment in multiple myeloma and goals for future clarity.	Blood Rev	2021	Excluded	Educational review — no original cohort, no MRD or PET-related survival outcomes	NO	Data on timing, methods, risk stratification, or details on imaging reading.	NO
38070713	Management and Outcomes of Patients with Refractory Solitary Plasmacytoma after Treatment with Definitive Radiation Therapy.	Int J Radiat Oncol Biol Phys	2024	Excluded	Wrong Disease	NO	Excluded at abstract step	NO
36878906	Integrated analysis of next generation sequencing minimal residual disease (MRD) and PET scan in transplant eligible myeloma patients.	Blood Cancer J	2023	Included	Meets all inclusion criteria	YES	Included	YES
36143236	Steps towards a Multiple Myeloma Cure?	J Pers Med	2022	Excluded	Educational review — no original cohort, no MRD or PET-related survival outcomes	NO	Excluded at abstract step	NO
31162104	International myeloma working group consensus recommendations on imaging in monoclonal plasma cell disorders.	Lancet Oncol	2019	Excluded	Review article or guideline, no original patient data	NO	Excluded at abstract step	NO
37629558	Current and Future PET Imaging for Multiple Myeloma.	Life (Basel)	2023	Excluded	Consensus/guideline paper — no original data or outcome measures	NO	Excluded at abstract step	NO
31024917	Interest of Pet Imaging in Multiple Myeloma.	Front Med (Lausanne)	2019	Excluded	Educational review — no original cohort, no MRD or PET-related survival outcomes	NO	Excluded at abstract step	NO
32751375	Functional Imaging for Therapeutic Assessment and Minimal Residual Disease Detection in Multiple Myeloma.	Int J Mol Sci	2020	Excluded	Educational review — no original cohort, no MRD or PET-related survival outcomes	NO	Excluded at abstract step	NO
37591548	New Developments in Myeloma Treatment and Response Assessment.	J Nucl Med	2023	Excluded	Technical/methods development paper — lacks	NO	Excluded at abstract step	NO

					clinical outcomes and MRD correlation			
31084778	Fludeoxyglucose F 18 PET/Computed Tomography Evaluation of Therapeutic Response in Multiple Myeloma.	PET Clin	2019	Excluded	Educational review — no original cohort, no MRD or PET-related survival outcomes	NO	Excluded at abstract step	NO
34363522	Imaging of treatment response and minimal residual disease in multiple myeloma: state of the art WB-MRI and PET/CT.	Skeletal Radiol	2022	Excluded	Review article or guideline, no original patient data	NO	Excluded at abstract step	NO
27556189	Application of PET/CT in treatment response evaluation and recurrence prediction in patients with newly-diagnosed multiple myeloma.	Oncotarget	2017	Maybe	Moved to 'Maybe' - unclear mention of MRD and PET in abstract	YES	PET has no PFS, OS Data. No MRD Data	NO
31447337	[Multiple myeloma: New criteria for diagnosis and treatment, strong therapeutic hopes].	Presse Med	2019	Excluded	Educational review — no original cohort, no MRD or PET-related survival outcomes	NO	Excluded at abstract step	NO
37509348	Minimal Residual Disease in Multiple Myeloma: Past, Present, and Future	Cancers (Basel)	2023	Excluded	Comprehensive review — no cohort data, no survival metrics, no dual PET/MRD evaluation	NO	Excluded at abstract step	NO
35190200	Modern radiographic imaging in multiple myeloma, what is the minimum requirement?	Semin Oncol	2022	Excluded	Technical/methods development paper — lacks clinical outcomes and MRD correlation	NO	Excluded at abstract step	NO
39355637	(18) F-FDG PET/CT and MRI in the Management of Multiple Myeloma: A Comparative Review.	Front Nucl Med	2021	Excluded	Review article or guideline, no original patient data	NO	Excluded at abstract step	NO
26877786	New Perspectives Offered by Nuclear Medicine for the Imaging and Therapy of Multiple Myeloma.	Theranostics	2016	Excluded	Editorial - no original patient data, no survival outcomes	NO	Excluded at abstract step	NO
37155114	Radiomics analysis of bone marrow biopsy locations in [(18)F]FDG PET/CT	Phys Eng Sci Med	2023	Excluded	No survival outcome reported (no PFS, OS, or TTNT); study focuses on correlation	NO	Excluded at abstract step	NO

	images for measurable residual disease assessment in multiple myeloma.				between PET features and MRD, not prognosis			
36044803	Bone marrow segmentation and radiomics analysis of [(18)F]FDG PET/CT images for measurable residual disease assessment in multiple myeloma.	Comput Methods Programs Biomed	2022	Excluded	Methods or biomarker correlation study, no survival outcomes	NO	Excluded at abstract step	NO
32645952	Minimal Residual Disease in Multiple Myeloma: State of the Art and Future Perspectives.	J Clin Med	2020	Excluded	Educational review — no original cohort, no MRD or PET-related survival outcomes	NO	Excluded at abstract step	NO
37328681	Dual assessment with multiparameter flow cytometry and (18)F-FDG PET/CT scan provides enhanced prediction of measurable residual disease after autologous haemopoietic stem cell transplant in myeloma-a prospective study.	Bone Marrow Transplant	2023	Maybe	Moved to 'Maybe' - unclear mention of MRD and PET in abstract	YES	Included	YES
31137045	Multiple Myeloma Guidelines and Their Recent Updates: Implications for Imaging.	Rofo	2019	Excluded	Educational review — no original cohort, no MRD or PET-related survival outcomes	NO	Excluded at abstract step	NO
34616538	Case series: MRD negativity assessment using (11)C-Acetate PET with 3-weekly daratumumab-based quadruplet induction in newly diagnosed multiple myeloma.	Ther Adv Hematol	2021	Excluded	Case Report	NO	Excluded at abstract step	NO
37568010	Intensifying treatment in PET-positive multiple myeloma patients after upfront autologous stem cell transplantation.	Leukemia	2023	Included	Meets all inclusion criteria	YES	Included	YES
32076595	Clinical Applications and Future Directions of	Front Oncol	2020	Excluded	Educational review — no original cohort, no MRD or	NO	Excluded at abstract step	NO

	Minimal Residual Disease Testing in Multiple Myeloma.				PET-related survival outcomes			
31283452	Whole-Body Imaging of Multiple Myeloma: Diagnostic Criteria.	Radiographics	2019	Excluded	Educational review — no original cohort, no MRD or PET-related survival outcomes	NO	Excluded at abstract step	NO
27696260	Multiple Myeloma Minimal Residual Disease.	Cancer Treat Res	2016	Excluded	Educational review — no original cohort, no MRD or PET-related survival outcomes	NO	Excluded at abstract step	NO
25754350	Is this the time to introduce minimal residual disease in multiple myeloma clinical practice?	Clin Cancer Res	2015	Excluded	Review article or guideline, no original patient data	NO	Excluded at abstract step	NO
29759149	Functional Imaging Methods for Assessment of Minimal Residual Disease in Multiple Myeloma: Current Status and Novel ImmunoPET Based Methods.	Semin Hematol	2018	Excluded	Educational review — no original cohort, no MRD or PET-related survival outcomes	NO	Excluded at abstract step	NO
37663672	Measurable Residual Disease Testing in Multiple Myeloma Routine Clinical Practice: A Modified Delphi Study.	Hemasphere	2023	Excluded	Educational review — no original cohort, no MRD or PET-related survival outcomes	NO	Excluded at abstract step	NO
30573775	Combination of flow cytometry and functional imaging for monitoring of residual disease in myeloma.	Leukemia	2019	Included	Meets all inclusion criteria	YES	No PFS, no OS Data	NO
38969566	Symptomatic Myeloma: PET, Whole-Body MR Imaging with Diffusion-Weighted Imaging or Both.	PET Clin	2024	Excluded	Educational review — no original cohort, no MRD or PET-related survival outcomes	NO	Excluded at abstract step	NO
31905752	Positron Emission Tomography (PET) Radiopharmaceuticals in Multiple Myeloma.	Molecules	2019	Excluded	Educational review — no original cohort, no MRD or PET-related survival outcomes	NO	Excluded at abstract step	NO

34921323	[Positron emission tomography/computed tomography (PET/CT) in multiple myeloma].	Radiologe	2022	Excluded	Review article or guideline, no original patient data	NO	Excluded at abstract step	NO
36579605	Functional Imaging in the Evaluation of Treatment Response in Multiple Myeloma: The Role of PET-CT and MRI.	J Pers Med	2022	Excluded	Educational review — no original cohort, no MRD or PET-related survival outcomes	NO	Excluded at abstract step	NO
30697462	FDG PET imaging in multiple myeloma: implications for response assessments in clinical trials.	Am J Nucl Med Mol Imaging	2018	Excluded	Case Report	NO	Excluded at abstract step	NO
40124904	Diagnostic Innovations: Advances in imaging techniques for diagnosis and follow-up of multiple myeloma.	J Bone Oncol	2025	Excluded	Narrative review article — no original patient data, no survival outcomes	NO	Excluded at abstract step	NO
35532876	Progress of modern imaging modalities in multiple myeloma.	Int J Hematol	2022	Excluded	Educational review — no original cohort, no MRD or PET-related survival outcomes	NO	Excluded at abstract step	NO
27249748	The Role of Imaging in the Treatment of Patients With Multiple Myeloma in 2016.	Am Soc Clin Oncol Educ Book	2016	Excluded	Educational review — no original cohort, no MRD or PET-related survival outcomes	NO	Excluded at abstract step	NO
32139013	Current and potential applications of positron emission tomography for multiple myeloma and plasma cell disorders.	Best Pract Res Clin Haematol	2020	Excluded	Educational review — no original cohort, no MRD or PET-related survival outcomes	NO	Excluded at abstract step	NO
33813607	Role of FDG PET in the staging of multiple myeloma.	Skeletal Radiol	2022	Excluded	Educational review – no original cohort, no MRD or PET-related survival outcomes	NO	Excluded at abstract step	NO
36382911	Carbon-11-Labeled Methionine PET/CT in Patients With FDG-Occult Multiple Myeloma: A Prospective Pilot Study.	AJR Am J Roentgenol	2023	Excluded	Moved to 'Maybe' - unclear mention of MRD and PET in abstract	YES	Pilot Study	NO

37657154	Evaluation of a semi-automated approach for FDG PET image analysis for routine clinical application in patients with multiple myeloma.	Transl Oncol	2023	Excluded	Technical/methods development paper – lacks clinical outcomes and MRD correlation	NO	Excluded at abstract step	NO
34938662	Early Dynamics and Depth of Response in Multiple Myeloma Patients Treated With BCMA CAR-T Cells.	Front Oncol	2021	Maybe	Moved to 'Maybe' - unclear mention of MRD and PET in abstract	YES	Descriptive concordance analysis with outcome data, estimated concordance group sizes from KM curves. No numeric HR	NO
29179894	[Preventative and therapeutic relapse strategies after allogeneic hematopoietic stem cell transplantation: Guidelines from the Francophone society of bone marrow transplantation and cellular therapy (SFGM-TC)].	Bull Cancer	2017	Excluded	Review article or guideline, no original patient data	NO	Excluded at abstract step	NO
33920809	Comparison of [(18)F]FDG PET/CT and MRI for Treatment Response Assessment in Multiple Myeloma: A Meta-Analysis.	Diagnostics (Basel)	2021	Maybe	Moved to 'Maybe' - unclear mention of MRD and PET in abstract	YES	No MRD reported; only PET/CT vs. MRI comparison for treatment response. No concordance analysis with MRD; comparison only between imaging modalities.	NO
33151787	Standardization of (18)F-FDG-PET/CT According to Deauville Criteria for Metabolic Complete Response Definition in Newly Diagnosed Multiple Myeloma.	J Clin Oncol	2021	Included	Meets all inclusion criteria	YES	Included	YES
37396807	Impact of minimal residual disease standardised assessment by FDG-PET/CT in transplant-eligible patients with newly diagnosed multiple myeloma enrolled in the imaging sub-study of the FORTE trial.	EClinicalMedicine	2023	Included	Meets all inclusion criteria	YES	Included	YES

33142671	Role of Imaging in the Evaluation of Minimal Residual Disease in Multiple Myeloma Patients.	J Clin Med	2021	Excluded	Review article or guideline, no original patient data	NO	Excluded at abstract step	NO
27409577	Multiple myeloma: disease response assessment.	Expert Rev Hematol	2016	Excluded	Educational review — no original cohort, no MRD or PET-related survival outcomes	NO	Excluded at abstract step	NO
30334460	Prognostic factors for multiple myeloma in the era of novel therapies.	Expert Rev Hematol	2018	Excluded	Educational review — no original cohort, no MRD or PET-related survival outcomes	NO	Excluded at abstract step	NO

B) Cochrane Central

CENTRAL_ID	Title	Source	Year	Abstract Analysis (Inclusion, Exclusion, Maybe)	Reason	Full Text Needed	Full Text Review	Included
CN-01028835	Comparison of mri and 18F-FDG pet for evaluation of the response to treatment of patients diagnosed with multiple myeloma: a prospective clinical study	Blood	2012	Maybe	Mentions only PET or MRD in abstract – full text needed to verify	NO	Out of Time Scope	NO
CN-01303249	Intensification therapy with bortezomib-melphalan-prednisone versus autologous stem cell transplantation for newly diagnosed multiple myeloma: an intergroup, multicenter, phase III study of the European myeloma network (EMN02/HO95 MM trial)	Blood	2016	Included	Mentions both PET and MRD in abstract	YES	No PET Data repoted	NO
CN-02051570	Evaluation of the prognostic value of positron emission tomography-computed tomography (PET-CT) at diagnosis and follow-up in transplant-eligible newly diagnosed multiple myeloma (TE NDMM) patients treated in the phase 3 cassiopeia study: results of the cassiopet companion study	Blood	2019	Included	Mentions both PET and MRD in abstract	YES	Duplicate	NO
CN-02295845	MRD Evaluation By PET/CT According to Deauville Criteria Combined with Multiparameter Flow Cytometry in Newly Diagnosed Transplant Eligible Multiple Myeloma (MM) Patients	Blood	2019	Included	Mentions both PET and MRD in abstract	YES	Duplicate	NO

	Enrolled in the Phase II Randomized Forte Trial							
CN-02258945	Impact of imaging FDG-PET/CT minimal residual disease assessment on outcomes and matching with bone marrow techniques in newly diagnosed transplant eligible multiple myeloma (MM) patients: results of the phase II randomized forte trial	Blood	2020	Included	Mentions both PET and MRD in abstract	YES	Duplicate	NO
CN-02258743	Treatment of high risk (HR) smoldering multiple myeloma (SMM) with carfilzomib, lenalidomide, and dexamethasone (KRD) followed by lenalidomide maintenance (-R): a phase 2 clinical and correlative study	Blood	2020	Included	Mentions both PET and MRD in abstract	YES	Data about Smolderng MM, not MM	NO
CN-02659547	Isatuximab in Combination with Lenalidomide and Dexamethasone in Patients with High-Risk Smoldering Multiple Myeloma: updated Safety Run-in Results from the Randomized Phase 3 Ithaca Study	Blood	2022	Included	Mentions both PET and MRD in abstract	YES	No PET Data repoted	NO
CN-02688265	CAR+ T-Cell Lymphoma Post Ciltacabtagene Autoleucel Therapy for Relapsed Refractory Multiple Myeloma	Blood	2023	Included	Mentions both PET and MRD in abstract	YES	No PET Data repoted	NO
CN-02668514	MRD Monitoring By Euroclonality IGH Based NGS Approach Predicts Outcome in Follicular Lymphoma Patients Lacking a Conventional BCL2:: IGH Marker: a Substudy from the Fondazione Italiana Linfomi (FIL) FOLL12 Trial	Blood	2023	Included	Mentions both PET and MRD in abstract	YES	No PET Data repoted	NO
CN-02664511	Will Survival Improve By Treating Multiple Myeloma	Blood	2023	Included	Mentions both PET and MRD in abstract	YES	Insufficient Data to be included	NO

	Patients at MRD Relapse? the Remnant Study							
CN-02244090	Evaluation of prognostic value of positron emission tomography-computed tomography (PET/CT) in transplant-eligible newly diagnosed multiple myeloma (NDMM) phase 3 cassiopeia study patients: cassiopet study results	Bone marrow transplantation	2020	Included	Mentions both PET and MRD in abstract	YES	Duplicate	NO
CN-01999614	Interim Analysis of Indian Multicentre Phase II Randomized Study Comparing Three Subcutaneous Bortezomib-based Post Stem Cell Transplantation Consolidation/ Maintenance Regimens for Newly Diagnosed Multiple Myeloma Patients (IMPOSe-Bortecon) Study Number: 4905/2017	Clinical lymphoma, myeloma & leukemia	2019	Included	Mentions both PET and MRD in abstract	YES	No PET Data repoted	NO
CN-02342761	P-137: ITHACA, a randomized multicenter phase 3 study of Isatuximab in combination with Lenalidomide and Dexamethasone in high-risk smoldering Multiple Myeloma: safety run-in preliminary results	Clinical lymphoma, myeloma & leukemia	2021	Maybe	Mentions only PET or MRD in abstract – full text needed to verify	YES	Study still running, no data on OS PFS or PET	NO
CN-02621679	Impact of minimal residual disease standardised assessment by FDG-PET/CT in transplant-eligible patients with newly diagnosed multiple myeloma enrolled in the imaging sub-study of the FORTE trial	EClinicalMedicine	2023	Included	Mentions both PET and MRD in abstract	YES	Duplicate	NO
CN-01106501	Therapeutic perspectives in smoldering multiple myeloma	Haematologica	2015	Included	Mentions both PET and MRD in abstract	YES	No PET Data repoted	NO
CN-01399192	Long term CR multiple myeloma patients studied with next generation flow show	Haematologica	2017	Included	Mentions both PET and MRD in abstract	YES	No PET Data repoted	NO

	predominantly cured VSmg us-like minimal residual disease patterns: a study of the GTMM-tuscan group for multiple myeloma							
CN-02009617	Standardization of 18F-FDG PET/CT according to deauville criteria for MRD evaluation in newly diagnosed transplant eligible multiple myeloma (MM) patients: joined analysis of two prospective randomized phase III trials	Haematologica	2019	Included	Mentions both PET and MRD in abstract	YES	Duplicate	NO
CN-02689240	IMPACT OF IMAGING FDG PET/CT MINIMAL RESIDUAL DISEASE ASSESSMENT ON OUTCOMES AND COMPLEMENTARITY WITH MULTIPARAMETER FLOW CYTOMETRY IN NEWLY DIAGNOSED TRANSPLANT ELIGIBLE MULTIPLE MYELOMA (MM) PATIENTS ENROLLED IN THE PHASE II RANDOMIZED FORTE TRIAL	Haematologica	2021	Included	Mentions both PET and MRD in abstract	YES	Duplicate	NO
CN-01792017	23rd European Hematology Association Congress	Hemasphere	2018	Included	Mentions both PET and MRD in abstract	YES	Meeting Report, no original Data	NO
CN-02144562	MRD evaluation by PET/CT according to deauville criteria combined with bone marrow techniques in newly diagnosed transplant eligible multiple myeloma patients enrolled in the phase ii forte trial	Hemasphere	2020	Included	Mentions both PET and MRD in abstract	YES	Duplicate	NO
CN-02461809	WILL OUTCOME IMPROVE BY TREATING MULTIPLE MYELOMA PATIENTS AT MRD RELAPSE? THE REMNANT STUDY (RELAPSE FROM MRD NEGATIVITY AS INDICATION FOR TREATMENT)	Hemasphere	2022	Included	Mentions both PET and MRD in abstract	YES	Study still running, no data on OS PFS or PET	NO

CN-01931939	Testing the Addition of Ixazomib/Placebo to Lenalidomide in Patients With Evidence of Residual Multiple Myeloma, OPTIMUM Trial	https://clinicaltrials.gov/ct2/show/NCT03941860	2019	Included	Mentions both PET and MRD in abstract	YES	Trial Registration Record. No Results	NO
CN-02555773	Alternate Doses and Dosing Schedules of Belantamab Mafodotin for Treatment of Triple-Class Refractory Multiple Myeloma	https://clinicaltrials.gov/ct2/show/NCT05847569	2023	Included	Mentions both PET and MRD in abstract	YES	Trial Registration Record. No Results	NO
CN-02633736	Comparing the Combination of Selinexor-Daratumumab-Velcade-Dexamethasone (Dara-SVD) With the Usual Treatment (Dara-RVD) for High-Risk Newly Diagnosed Multiple Myeloma	https://clinicaltrials.gov/ct2/show/NCT06169215	2023	Included	Mentions both PET and MRD in abstract	YES	Trial Registration Record. No Results	NO
CN-02635446	Iberdomide Versus Observation Off Therapy After Idecabtagene Vicleucel CAR-T for Multiple Myeloma	https://clinicaltrials.gov/ct2/show/NCT06179888	2023	Included	Mentions both PET and MRD in abstract	YES	Trial Registration Record. No Results	NO
CN-02679912	Testing the Combination of Two Approved Drugs and One Experimental Drug in Patients With Relapsed or Refractory Multiple Myeloma	https://clinicaltrials.gov/ct2/show/NCT06232044	2024	Included	Mentions both PET and MRD in abstract	YES	Trial Registration Record. No Results	NO
CN-01794930	Treatment for Elderly Fit Newly Diagnosed Multiple Myeloma Patients Aged Between 65 and 80 Years	https://clinicaltrials.gov/show/NCT03742297	2018	Included	Mentions both PET and MRD in abstract	YES	Trial Registration Record. No Results	NO
CN-01983022	Lenalidomide, and Dexamethasone With or Without Daratumumab in Treating Patients With High-Risk Smoldering Myeloma	https://clinicaltrials.gov/show/NCT03937635	2019	Included	Mentions both PET and MRD in abstract	YES	Trial Registration Record. No Results	NO
CN-02181506	Testing the Use of Combination Therapy in Adult Patients With Newly Diagnosed Multiple Myeloma, the EQUATE Trial	https://clinicaltrials.gov/show/NCT04566328	2020	Included	Mentions both PET and MRD in abstract	YES	Trial Registration Record. No Results	NO
CN-02786452	A phase 1/2, dose and schedule evaluation study to investigate the safety and clinical activity of Belantamab	https://trialsearch.who.int/Trial2.aspx?TrialID=CT	2024	Included	Mentions both PET and MRD in abstract	YES	Trial Registration Record. No Results	NO

	Mafodotin administered in combination with Lenalidomide and Dexamethasone in patients with newly diagnosed multiple myeloma translplant ineligible	IS2024-515988-55-00						
CN-01947417	A MULTICENTER, OPEN LABEL, RANDOMIZED PHASE II STUDY COMPARING DARATUMUMAB combined with BORTEZOMIB-CYCLOPHOSPHAMIDE-DEXAMETHASONE (Dara-VCd) VERSUS THE ASSOCIATION OF BORTEZOMIB-THALIDOMIDE-DEXAMETHASONE (VTd) AS PRE TRANSPLANT INDUCTION AND POST TRANSPLANT CONSOLIDATION, BOTH FOLLOWED BY A MAINTENANCE PHASE WITH IXAZOMIB ALONE OR IN COMBINATION WITH DARATUMUMAB, IN NEWLY DIAGNOSED MULTIPLE MYELOMA (MM) YOUNG PATIENTS eligible for AUTOLOGOUS STEM CELL TRANSPLANTATION	https://trialsearch.who.int/Trial2.aspx?TrialID=EUCTR2018-002089-37-GR	2018	Included	Mentions both PET and MRD in abstract	YES	Trial Registration Record. No Results	NO
CN-01948076	A Study of combination of Daratumumab, VELCADE (bortezomib), Lenalidomide, and Dexamethasone (D-VRd) compared to VELCADE, Lenalidomide, and Dexamethasone (VRd) in participants with Previously Untreated Multiple Myeloma	https://trialsearch.who.int/Trial2.aspx?TrialID=EUCTR2018-002992-16-GR	2018	Included	Mentions both PET and MRD in abstract	YES	Trial Registration Record. No Results	NO
CN-02255626	Multicenter Open label Phase 3 study of Isatuximab plus Lenalidomide and Dexamethasone with/without Bortezomib in the Treatment of Newly diagnosed Non Frail transplant Ineligible Multiple	https://trialsearch.who.int/Trial2.aspx?TrialID=EUCTR2020-004602-59-FR	2020	Included	Mentions both PET and MRD in abstract	YES	No PET Data	NO

	Myeloma elderly patients (= 65; < 80 years)							
CN-02257036	Belantamab Mafodotin in combination with Lenalidomide and Dexamethasone for the treatment of patients with newly diagnosed multiple myeloma who transplant ineligible	https://trialsearch.who.int/Trial2.aspx?TrialID=EUCTR2020-005826-27-GR	2021	Included	Mentions both PET and MRD in abstract	YES	Insufficient Data to be included	NO
CN-02410507	Belantamab Mafodotin in combination with Daratumumab, Lenalidomide and Dexamethasone for the treatment of patients with newly diagnosed multiple myeloma transplant ineligible	https://trialsearch.who.int/Trial2.aspx?TrialID=EUCTR2021-006792-42-GR	2022	Included	Mentions both PET and MRD in abstract	YES	Insufficient Data to be included	NO
CN-02593092	A phase 1/2, dose and schedule evaluation study to investigate the safety and clinical activity of belantamab mafodotin administered in combination with lenalidomide, dexamethasone and nirogacestat in patients with transplant ineligible newly diagnosed multiple myeloma	https://trialsearch.who.int/Trial2.aspx?TrialID=EUCTR2022-001942-39-GR	2022	Included	Mentions both PET and MRD in abstract	YES	Insufficient Data to be included	NO
CN-01948638	A Study Comparing Daratumumab, VELCADE (bortezomib), Lenalidomide, and Dexamethasone (D-VRd) with VELCADE, Lenalidomide, and Dexamethasone (VRd) in Participants with Untreated Multiple Myeloma and for Whom Hematopoietic Stem Cell Transplant is Not Planned as Initial Therapy	https://trialsearch.who.int/Trial2.aspx?TrialID=JPRN-JapicCTI-184162	2018	Included	Mentions both PET and MRD in abstract	YES	Trial Registration Record. No Results	NO
CN-02241959	JCOG1911: randomized phase III study of daratumumab (D) versus bortezomib plus D as a maintenance therapy after D-	https://trialsearch.who.int/Trial2.aspx?TrialID=JP	2021	Included	Mentions both PET and MRD in abstract	YES	Trial Registration Record. No Results	NO

	MPB for Elderly or non-elderly patients refusing transplant with untreated multiple myeloma	RN-jRCTs031200320						
CN-02719917	A Phase 3 Study Comparing Daratumumab, VELCADE (bortezomib), Lenalidomide, and Dexamethasone (D-VRd) vs VELCADE, Lenalidomide, and Dexamethasone (VRd) in Subjects with Previously Untreated Multiple Myeloma who are Eligible for High-dose Therapy	https://trialsearch.who.int/Trial2.aspx?TrialID=NL-OMON52378	2018	Included	Mentions both PET and MRD in abstract	YES	Trial Registration Record. No Results	NO
CN-02511096	Indian Multicenter Phase Ii Randomised Controlled Study Comparing Post Stem Cell Maintenance Regimen for Newly Diagnosed Multiple Myeloma (Impose Bortecon Study)	Indian journal of hematology & blood transfusion	2022	Included	Mentions both PET and MRD in abstract	YES	Trial Registration Record. No Results	NO
CN-01397394	Phase 2, open-label study of venetoclax in combination with carfilzomib and dexamethasone in patients with relapsed/refractory multiple myeloma	Journal of clinical oncology	2017	Included	Mentions both PET and MRD in abstract	YES	Trial Registration Record. No Results	NO
CN-02201778	Standardization of 18F-FDG-PET/CT According to Deauville Criteria for Metabolic Complete Response Definition in Newly Diagnosed Multiple Myeloma	Journal of clinical oncology	2021	Included	Mentions both PET and MRD in abstract	YES	Duplicate	NO

Supplementary Table S3. Per-study MRD×PET-CT concordance metrics and 2×2 counts

Study	n	MRD^{-}/PET-CT^{+}	MRD^{-}/PET-CT^{-}	MRD^{+}/PET-CT^{-}	MRD^{+}/PET-CT^{+}	pct_agree	Kappa	McNemar_p	AC1	AC1_se	AC1_lcl	AC1_ucl	PABAK	PABAK_lcl	PABAK_ucl
Alonso R et al 2019	103	13	56	28	6	60·2	-0·01	0·03	0·36	0.08	0·20	0·51	0·20	0·00	0·40
Moreau et al 2019	176	12	102	55	7	61·9	0·00	0·00	0·41	0·06	0·30	0·52	0·24	0·09	0·39
Zamagni E et al 2021	228	42	64	39	83	64·5	0·28	0·82	0·30	0·06	0·17	0·42	0·29	0·16	0·41
Broeckle D et al 2022	78	6	35	31	6	52·6	0·01	0·00	0·17	0·10	-0·03	0·36	0·05	-0·18	0·28
Zamagni E et al 2023	109	20	63	6	20	76·1	0·45	0·01	0·60	0·07	0·45	0·72	0·52	0·34	0·67
Mookerjee A et al 2023	131	14	78	31	8	65·6	0·061	0·02	0·46	0·06	0·34	0·60	0·31	0·14	0·47
Fonseca R et al 2023	136	2	32	79	23	40·4	0·09	0·00	-0·19	0·08	-0·35	-0·02	-0·19	-0·36	-0·01
Hajiyianni M et al 2024	72	6	27	28	11	52·8	0·09	0·00	0·10	0·11	-0·12	0·32	0·05	-0·19	0·30
Swain et al 2025	82	30	25	8	19	53·7	0·13	0·00	0·08	0·11	-0·14	0·30	0·07	-0·15	0·29
Talarico et al 2025	23	0	17	5	1	78·3	0·23	0·074	0·70	0·12	0·48	0·94	0·56	0·13	0·85

Study-level 2×2 counts (a = MRD^{-}/PET-CT^{+}; b = MRD^{-}/PET-CT^{-}; c = MRD^{+}/PET-CT^{-}; d = MRD^{+}/PET-CT^{+}) and concordance metrics at the primary landmark. pct_agree = observed percent agreement (b + d)/n· κ = Cohen's kappa; AC1 = Gwet's AC1; PABAK = prevalence- and bias-adjusted κ. McNemar_p = exact p value for asymmetry of discordant cells (a vs c). Confidence intervals (AC1_lcl/ucl and PABAK_lcl/ucl) are 95% Cis. These values correspond to the primary concordance analyses reported in Figure 2 and the main text.

Supplementary Table S4. Pooled MRD × PET-CT 2×2 counts and agreement (final analytic set)

Dataset	*a*	*b*	*c*	*d*	N	Agreeement (%)
Primary	145	499	310	184	1138	0·600
Strict Timepoint	127	370	166	227	890	0·672

Pooled joint MRD × PET-CT counts and observed agreement for the primary and strict-pairing ($\Delta \leq 30$ days) definitions· a = $MRD^{-}/PET\text{-}CT^{+}$; b = $MRD^{-}/PET\text{-}CT^{-}$; c = $MRD^{+}/PET\text{-}CT^{-}$; d = $MRD^{+}/PET\text{-}CT^{+}$; N = total number of paired assessments· Observed agreement is defined as (b + d)/N and is shown as a proportion and percentage. Counts and N correspond to the final analytic dataset used in the main concordance analyses

Table S5. Fréchet–Hoeffding bounds and identifiability class for κ (per study)

Study	n	K_point	K_low	K_high	Width	Class
Alonso R et al 2019	103	-0·01	-0·31	0·63	0·94	Non-identified
Moreau et al 2019	176	0·01	-0·19	0·36	0·56	Non-identified
Zamagni E et al 2021	228	0·29	-0·84	0·97	1·82	Non-identified
Broeckle D et al 2022	78	0·02	-0·30	0·33	0·64	Non-identified
Zamagni E et al 2023	109	0·45	-0·40	0·70	1·10	Non-identified
Mookerjee A et al 2023	131	0·06	-0·27	0·64	0·92	Non-identified
Fonseca R et al 2023	136	0·10	-0·42	0·14	0·56	Non-identified
Hajiyianni M et al 2024	72	0·10	-0·49	0·41	0·90	Non-identified
Swain et al 2025	82	0·13	-0·74	0·50	1·24	Non-identified
Talarico et al 2025	23	0·23	-0·08	0·23	0·31	Non-identified

Bounds on Cohen's κ implied by the reported MRD and PET-CT margins without assuming independence of discordant cells. Width = K_high − K_low. "Class" indicates whether κ is point-identified as positive or negative or only bounded (non-identified) based on the FH interval; in this dataset, all studies are classified as non-identified because their FH intervals cross 0.

Supplementary Table S6. Between-study heterogeneity estimators for the PFS meta-analysis

Model	k	HR (95% CI)	τ^2	I^2 (%)
REML (Knapp–Hartung)	5	0·34 (0·22–0·51)	0·03	26·72
Paule–Mandel (Knapp–Hartung)	5	0·33 (0·22–0·51)	0·03	24·89
Sidik–Jonkman (Knapp–Hartung)	5	0·34 (0·22–0·52)	0·06	42·78

Comparison of τ^2 and I^2 across REML, Paule–Mandel and Sidik–Jonkman estimators, with pooled HRs (Knapp–Hartung inference). τ^2 = between-study variance; I^2 = heterogeneity; HR = hazard ratio; τ^2 estimators = REML, Paule–Mandel, Sidik–Jonkman.

Supplementary Table S7. PFS meta-analysis: per-study hazard ratios and model inputs

Study	logHR	SE	vi	HR	HR (95% CI) LCL	HR (95% CI) UCL
Alonso R, et al.	-1·51	0·24	0·06	0·22	0·14	0·35
Kraeber F, et al	-0·94	0·27	0·07	0·39	0·23	0·67
Zamagni E, et al.	-0·8	0·34	0·12	0·45	0·23	0·88
Mookerjee A, et al	-1·15	0·23	0·05	0·32	0·2	0·5
Fonseca R, et al.	-0·56	0·48	0·23	0·57	0·22	1·47

Study-level log(hazard ratio) (logHR), standard error (SE), within-study variance (vi), and hazard ratios (HR) with lower and upper 95% confidence limits (HR 95% CI LCL, HR 95% CI UCL) for dual-negative versus all other MRD/PET-CT categories. These values are used in the random-effects PFS meta-analysis (Figure 3). PFS = progression-free survival; HR = hazard ratio; CI = confidence interval.

Supplementary Table S8. Leave-one-out and sensitivity meta-analyses (Strict pairing; high-risk excluded)

Sensitivity set	k	HR	HR (95% CI) LCL	HR (95% CI) UCL	Notes
All studies (baseline, STRICT pairing)	5				Reference pooled effect under strict pairing (Δ ≤ 30 d).
Leave-one-out: Alonso et al omitted	4	0·38	0·28	0·51	Equivalent to "High-risk (QUIPS overall High) excluded."
Leave-one-out: Zamagni et al omitted	4	0·30	0·19	0·46	
Leave-one-out: Fonseca et al omitted	4	0·30	0·21	0·44	
Leave-one-out: Mookerjee et al omitted	4	0·35	0·20	0·62	
Leave-one-out: Kraeber et al omitted	4	0·33	0·22	0·47	
High-risk (QUIPS overall High) excluded	4	0·38	0·28	0·51	Only Alonso 2019 is Overall High on QUIPS; identical to "Alonso et al omitted."

Pooled hazard ratios (HRs) and 95% confidence intervals (HR 95% CI LCL, HR 95% CI UCL) for progression-free survival comparing dual-negative versus all other MRD/PET-CT categories, restricted to strictly paired assessments (Δ ≤ 30 days or simultaneous). Rows show the baseline model including all studies (k = 5), leave-one-out deletions (k = 4), and the prespecified sensitivity excluding studies at Overall High QUIPS risk of bias. Because only Alonso et al 2019 was rated Overall High on QUIPS in the prognostic set, the "High-risk excluded" sensitivity is numerically identical to the "Alonso et al omitted" row. All estimates are from random-effects models (REML) with Hartung–Knapp inference.

Supplementary Table S9. Risk-of-bias matrices per study (QUADAS-2 for concordance; QUIPS for prognosis).

Per-study judgements for each QUADAS-2 (concordance) and QUIPS (prognosis) domain and overall rating, aligned to the datasets used in Figures 2–3 and Tables S3–S7. QUADAS-2 = Quality Assessment of Diagnostic Accuracy Studies-2; QUIPS = Quality in Prognosis Studies.

QUIPS

Study	Domain	Judgement
Alonso R et al 2019	Outcome measurement	Moderate
Alonso R et al 2019	Prognostic factor measurement	High
Alonso R et al 2019	Statistical analysis and reporting	Moderate
Alonso R et al 2019	Study attrition	Moderate
Alonso R et al 2019	Study confounding	Moderate
Alonso R et al 2019	Study participation	Moderate
Broeckle D et al 2022	Outcome measurement	Moderate
Broeckle D et al 2022	Prognostic factor measurement	Moderate
Broeckle D et al 2022	Statistical analysis and reporting	Low
Broeckle D et al 2022	Study attrition	Moderate
Broeckle D et al 2022	Study confounding	Low
Broeckle D et al 2022	Study participation	Moderate
Fonseca R et al 2023	Outcome measurement	Moderate
Fonseca R et al 2023	Prognostic factor measurement	Moderate
Fonseca R et al 2023	Statistical analysis and reporting	Low
Fonseca R et al 2023	Study attrition	Moderate
Fonseca R et al 2023	Study confounding	Moderate
Fonseca R et al 2023	Study participation	Moderate
Hajiyianni M et al 2024	Outcome measurement	Not Applicable
Hajiyianni M et al 2024	Prognostic factor measurement	Not Applicable
Hajiyianni M et al 2024	Statistical analysis and reporting	Not Applicable
Hajiyianni M et al 2024	Study attrition	Not Applicable
Hajiyianni M et al 2024	Study confounding	Not Applicable
Hajiyianni M et al 2024	Study participation	Not Applicable
Mookerjee A et al 2023	Outcome measurement	Moderate
Mookerjee A et al 2023	Prognostic factor measurement	Low
Mookerjee A et al 2023	Statistical analysis and reporting	Low
Mookerjee A et al 2023	Study attrition	Low
Mookerjee A et al 2023	Study confounding	Moderate
Mookerjee A et al 2023	Study participation	Low
Moreau et al 2019-CASSIOPET	Outcome measurement	Low
Moreau et al 2019-CASSIOPET	Prognostic factor measurement	Low
Moreau et al 2019-CASSIOPET	Statistical analysis and reporting	Low
Moreau et al 2019-CASSIOPET	Study attrition	Moderate

Moreau et al 2019-CASSIOPET	Study confounding	Low
Moreau et al 2019-CASSIOPET	Study participation	Moderate
Swain R et al 2025	Outcome measurement	Low
Swain R et al 2025	Prognostic factor measurement	Low
Swain R et al 2025	Statistical analysis and reporting	Moderate
Swain R et al 2025	Study attrition	Low
Swain R et al 2025	Study confounding	Moderate
Swain R et al 2025	Study participation	Moderate
Talarico M et al 2025	Outcome measurement	Not Applicable
Talarico M et al 2025	Prognostic factor measurement	Moderate
Talarico M et al 2025	Statistical analysis and reporting	Low
Talarico M et al 2025	Study attrition	Moderate
Talarico M et al 2025	Study confounding	High
Talarico M et al 2025	Study participation	High
Zamagni E et al 2021	Outcome measurement	Low
Zamagni E et al 2021	Prognostic factor measurement	Low
Zamagni E et al 2021	Statistical analysis and reporting	Low
Zamagni E et al 2021	Study attrition	Low
Zamagni E et al 2021	Study confounding	Low
Zamagni E et al 2021	Study participation	Low
Zamagni E et al 2023	Outcome measurement	Low
Zamagni E et al 2023	Prognostic factor measurement	Low
Zamagni E et al 2023	Statistical analysis and reporting	Low
Zamagni E et al 2023	Study attrition	Moderate
Zamagni E et al 2023	Study confounding	Low
Zamagni E et al 2023	Study participation	Moderate

QUADAS2

Study	Domain	Judgement
Alonso R et al 2019	Flow & timing	Some concerns
Alonso R et al 2019	Index test (MRD)	Some concerns
Alonso R et al 2019	Index test (PET)	Low
Alonso R et al 2019	Patient selection	Some concerns
Alonso R et al 2019	Reference standard	Some concerns
Broeckle D et al 2022	Flow & timing	Low
Broeckle D et al 2022	Index test (MRD)	Low
Broeckle D et al 2022	Index test (PET)	Some concerns
Broeckle D et al 2022	Patient selection	Low
Broeckle D et al 2022	Reference standard	High
Fonseca R et al 2023	Flow & timing	Low
Fonseca R et al 2023	Index test (MRD)	Some concerns
Fonseca R et al 2023	Index test (PET)	Some concerns
Fonseca R et al 2023	Patient selection	Some concerns
Fonseca R et al 2023	Reference standard	Some concerns
Hajiyianni M et al 2024	Flow & timing	Some concerns
Hajiyianni M et al 2024	Index test (MRD)	Some concerns
Hajiyianni M et al 2024	Index test (PET)	Some concerns
Hajiyianni M et al 2024	Patient selection	Some concerns
Hajiyianni M et al 2024	Reference standard	Some concerns
Mookerjee A et al 2023	Flow & timing	Some concerns
Mookerjee A et al 2023	Index test (MRD)	Low
Mookerjee A et al 2023	Index test (PET)	Low
Mookerjee A et al 2023	Patient selection	Low
Mookerjee A et al 2023	Reference standard	Some concerns
Moreau et al 2019-CASSIOPET	Flow & timing	Low
Moreau et al 2019-CASSIOPET	Index test (MRD)	Low
Moreau et al 2019-CASSIOPET	Index test (PET)	Some concerns
Moreau et al 2019-CASSIOPET	Patient selection	Low
Moreau et al 2019-CASSIOPET	Reference standard	High
Swain R et al 2025	Flow & timing	Low
Swain R et al 2025	Index test (MRD)	Some concerns
Swain R et al 2025	Index test (PET)	Some concerns
Swain R et al 2025	Patient selection	Some concerns
Swain R et al 2025	Reference standard	Some concerns
Talarico M et al 2025	Flow & timing	High
Talarico M et al 2025	Index test (MRD)	Some concerns
Talarico M et al 2025	Index test (PET)	Some concerns

Talarico M et al 2025	Patient selection	High
Talarico M et al 2025	Reference standard	Some concerns
Zamagni E et al 2021	Flow & timing	Low
Zamagni E et al 2021	Index test (MRD)	Low
Zamagni E et al 2021	Index test (PET)	Low
Zamagni E et al 2021	Patient selection	Low
Zamagni E et al 2021	Reference standard	Low
Zamagni E et al 2023	Flow & timing	Some concerns
Zamagni E et al 2023	Index test (MRD)	Low
Zamagni E et al 2023	Index test (PET)	Low
Zamagni E et al 2023	Patient selection	Low
Zamagni E et al 2023	Reference standard	Some concerns

Supplementary Table S10. Overall QUADAS-2 and QUIPS risk-of-bias classifications by study

Study	Quips overall	Quadas overall
Alonso R et al 2019	High	Some concerns
Broeckle D et al 2022	Moderate	High
Fonseca R et al 2023	Moderate	Some concerns
Hajiyianni M et al 2024	Low	Some concerns
Mookerjee A et al 2023	Moderate	Some concerns
Moreau et al 2019-CASSIOPET	Moderate	High
Swain R et al 2025	Moderate	Some concerns
Talarico M et al 2025	High	High
Zamagni E et al 2021	Low	Low
Zamagni E et al 2023	Moderate	Some concerns

Overall risk-of-bias judgements (Low, Moderate, High) for each study under QUADAS-2 (concordance) and QUIPS (prognosis), as used to define the "High-risk excluded" sensitivity analyses. These classifications correspond to the domain-level assessments shown in Figures S5 and S6.
